# Association Between Intensity of Low-Density Lipoprotein Cholesterol Reduction With Statin-Based Therapies and Secondary Stroke Prevention: A Meta-analysis of Randomized Clinical Trials

**DOI:** 10.1001/jamaneurol.2021.5578

**Published:** 2022-02-21

**Authors:** Meng Lee, Chun-Yu Cheng, Yi-Ling Wu, Jiann-Der Lee, Chia-Yu Hsu, Bruce Ovbiagele

**Affiliations:** 1 Department of Neurology, Chang Gung University College of Medicine, Chang Gung Memorial Hospital, Chiayi, Taiwan; 2 Department of Neurosurgery, Chang Gung University College of Medicine, Chang Gung Memorial Hospital, Chiayi, Taiwan; 3 Institute of Population Health Sciences, National Health Research Institutes, Miaoli County, Taiwan; 4 Department of Neurology, University of California, San Francisco

## Abstract

**Importance:**

The benefits and risks associated with intensive low-density lipoprotein cholesterol (LDL-C)–lowering statin-based therapies to lessen the risk of recurrent stroke have not been established.

**Objective:**

To conduct a meta-analysis of randomized clinical trials to evaluate the association of more intensive vs less intensive LDL-C–lowering statin-based therapies with outcomes for patients with ischemic stroke.

**Data Sources:**

PubMed, Embase, the Cochrane Central Register of Controlled Trials, and ClinicalTrials.gov were searched from January 1, 1970, to July 31, 2021.

**Study Selection:**

This meta-analysis included randomized clinical trials that compared more intensive vs less intensive LDL-C–lowering statin-based therapies and recorded the outcome of recurrent stroke among patients with stroke.

**Data Extraction and Synthesis:**

The Preferred Reporting Items for Systematic Reviews and Meta-analyses (PRISMA) reporting guideline was used for abstracting data and assessing data quality and validity. Relative risk (RR) with 95% CI was used as a measure of the association of more intensive vs less intensive LDL-C lowering with primary and secondary outcomes.

**Main Outcomes and Measures:**

The primary outcome was recurrent stroke, and the secondary outcomes were major cardiovascular events and hemorrhagic stroke.

**Results:**

The final analysis included 11 randomized clinical trials with 20 163 patients (13 518 men [67.0%]; mean [SD] age, 64.9 [3.7] years) with stroke. The mean follow-up was 4 years (range, 1-6.1 years). Pooled results showed that more intensive LDL-C–lowering statin-based therapies were associated with a reduced risk of recurrent stroke compared with less intensive LDL-C–lowering statin-based therapies (absolute risk, 8.1% vs 9.3%; RR, 0.88; 95% CI, 0.80-0.96) and that the benefit associated with these LDL-C–lowering therapies was not different among LDL-C–lowering strategies (statins vs no statins: RR, 0.90; 95% CI, 0.81-1.01; more statins or ezetimibe vs less statins or ezetimibe: RR, 0.77; 95% CI, 0.62-0.96; and proprotein convertase subtilisin/kexin type 9 inhibitors plus statins vs placebo plus statins: RR, 0.90; 95% CI, 0.71-1.15; *P* = .42 for interaction). More intensive LDL-C–lowering statin-based therapies were associated with a reduced risk of major cardiovascular events, but with an increased risk of hemorrhagic stroke, compared with less intensive LDL-C–lowering statin-based therapies. More intensive LDL-C–lowering statin-based therapies were associated with a reduced risk of recurrent stroke in trials with all patients having evidence of atherosclerosis (RR, 0.79; 95% CI, 0.69-0.91), but not in trials with most patients not having evidence of atherosclerosis (RR, 0.95; 95% CI, 0.85-1.07; *P* = .04 for interaction), compared with less intensive LDL-C–lowering statin-based therapies.

**Conclusions and Relevance:**

This study suggests that the benefits and risks of more intensive LDL-C–lowering statin-based therapies for recurrent stroke risk reduction might be more favorable than the benefits and risks of less intensive LDL-C–lowering statin-based therapies, especially for patients with evidence of atherosclerosis.

## Key Points

**Question:**

Are more intensive low-density lipoprotein cholesterol (LDL-C)–lowering statin-based therapies beneficial for secondary stroke prevention compared with less intensive LDL-C lowering?

**Findings:**

In this meta-analysis that included 11 randomized clinical trials comprising 20 163 patients with stroke, the risk of recurrent stroke was 8% with more intensive LDL-C lowering vs 9% with less intensive LDL-C lowering, a statistically significant difference. The benefits associated with more intensive LDL-C lowering might be found only in patients with ischemic stroke with evidence of atherosclerosis.

**Meaning:**

This study suggests that more intensive LDL-C–lowering statin-based therapies might be warranted for patients with ischemic stroke with evidence of atherosclerosis.

## Teaser

This meta-analysis of randomized clinical trials evaluates the association of more intensive vs less intensive low-density lipoprotein cholesterol–lowering statin-based therapies with outcomes for patients with ischemic stroke.

## Introduction

An elevated low-density lipoprotein cholesterol (LDL-C) level is a risk factor for cardiovascular disease, including ischemic stroke.^1^ For patients with a history of ischemic stroke, an elevated LDL-C level is associated with an increased risk of subsequent major cardiovascular events.^2^ More intensive compared with less intensive LDL-C–lowering statin-based therapies are associated with reduced major cardiovascular events in patients with established atherosclerotic cardiovascular disease.^3^ However, the results of LDL lowering with statins in secondary stroke prevention trials are inconsistent. An initial meta-analysis of randomized clinical trials showed that intensive LDL-C reduction with statins was associated with a significantly reduced risk of recurrent stroke.^4^ A subsequent meta-analysis of randomized clinical trials showed that statins were associated with a reduction in the risk of ischemic strokes and cardiovascular events, but the reduction of recurrent stroke did not reach statistical significance.^5^ In addition to their LDL-C–lowering effects, statins may exhibit cardiovascular protection via their pleiotropic effects.^6,7^ The antithrombotic effect of statins may provide additional reduction in ischemic events but may increase the risk of intracranial hemorrhage in patients with ischemic stroke.^7,8^

Statins plus cholesterol absorption inhibitors (eg, ezetimibe) or proprotein convertase subtilisin/kexin type 9 (PCSK9) inhibitors (alirocumab and evolocumab) compared with statins alone were associated with reduced major cardiovascular events and strokes for patients with a history of acute coronary syndrome or atherosclerotic cardiovascular disease in clinical trials.^9,10,11^ However, whether those medications (ezetimibe or PCSK9 inhibitors) are beneficial as add-on therapy to statins for patients with prior stroke has not been definitively established, to our knowledge.

To properly elucidate the association of LDL-C–lowering statin-based therapies with secondary stroke prevention, we conducted a systematic review and meta-analysis of randomized clinical trials to qualitatively and quantitatively evaluate the benefits and risks associated with more intensive vs less intensive LDL-C–lowering statin-based therapies for patients with ischemic stroke.

## Methods

The Preferred Reporting Items for Systematic Reviews and Meta-analyses (PRISMA) reporting guideline was used for abstracting data and validity of this meta-analysis.^12^ The protocol was registered with PROSPERO (CRD42020193206).

### Search Methods and Resources

We searched PubMed, Embase, the Cochrane Central Register of Controlled Trials, and the clinical trial registry maintained at ClinicalTrials.gov from January 1, 1970, to July 31, 2021, using the following terms: statins OR hydroxymethylglutaryl-CoA reductase inhibitors OR HMG-CoA reductase inhibitor OR HMG-CoA statins OR atorvastatin OR simvastatin OR fluvastatin OR pravastatin OR pitavastatin OR rosuvastatin OR lovastatin OR ezetimibe OR ezetrol OR vytorin OR bempedoic acid OR nilemdo OR nexletol OR proprotein convertase subtilisin/kexin type 9 inhibitor OR PCSK9 inhibitor OR alirocumab OR evolocumab AND stroke OR cerebrovascular accident OR brain vascular accident OR cerebrovascular stroke OR apoplexy OR cerebral infarct OR cerebrovascular disorder OR intracranial vascular disease OR cerebrovascular disease OR brain vascular disorder OR cerebrovascular occlusion OR cerebrovascular insufficiency. We limited search results to human studies and randomized clinical trials. We also reviewed the introduction and discussion sections of retrieved trials and of prior meta-analyses^3,4,5^ to identify additional trials.

### Study Selection and Data Extraction

Criteria for inclusion of a study were as follows: (1) the study design was a randomized clinical trial; (2) all or an identifiable subset of participants had a history of stroke or transient ischemic attack; (3) the study evaluated more intensive vs less intensive LDL-C–lowering statin-based therapies, including the following possible comparisons: statins vs no statins, more statins or ezetimibe vs less statins or ezetimibe (eg, more intensive statins vs less intensive statins; ezetimibe plus statins vs placebo plus statins), and PCSK9 inhibitors plus statins vs placebo plus statins; (4) recurrent stroke was reported as an end point; and (5) treatment duration was at least 6 months.

We excluded trials with more than 10% of participants having end-stage kidney disease because the clinical benefit associated with lipid-lowering therapy is confounded by competing nonatherosclerotic risks. One investigator (C.-Y.H.) abstracted the data, and another investigator (M.L.) reviewed the extracted data. Any discrepant judgments were resolved by joint discussion.

### Study Quality Assessment

Because all of the included studies were randomized clinical trials, the risk of bias (eg, selection bias, performance bias, detection bias, attrition bias, reporting bias, and other issues) of the included trials was assessed by the Cochrane risk-of-bias algorithm.^13,14^

### Statistical Analysis

The analysis plan was performed on an intention-to-treat basis. The primary outcome of interest was recurrent stroke. The secondary outcomes of interest were major adverse cardiovascular events (MACEs), recurrent ischemic stroke, hemorrhagic stroke, myocardial infarction, all-cause mortality, cardiovascular death, new-onset diabetes, and cognitive adverse events. A MACE was defined as a composite of cardiovascular death, nonfatal myocardial infarction, and nonfatal stroke or the nearest equivalent. Studies were categorized into 3 subgroups: statins vs no statins, more statins or ezetimibe vs less statins or ezetimibe, and PCSK9 inhibitors plus statins vs placebo plus statins. We computed the fixed-effects estimate based on the Mantel-Haenszel method when 2 or more studies provided sufficient data for a given outcome and compared the results with those obtained from the random-effects model. Relative risk (RR) with 95% CI was used as a measure of the association of more intensive vs less intensive LDL-C lowering with the primary and secondary outcomes. All *P* values were from 2-sided tests, and results were deemed statistically significant at *P* < .05. Heterogeneity was assessed by a *P* value determined by the use of χ^2^ statistics and *I*^2^ statistics, and *I*^2^ values of 0% to 29%, 30% to 49%, 50% to 74%, and 75% to 100% represent not important, moderate, substantial, and considerable inconsistency, respectively.^15^

Subgroup analyses based on the primary outcome were conducted according to different study characteristics: baseline LDL-C level (≥100 vs <100 mg/dL [to convert to millimoles per liter, multiply by 0.0259]), degree of LDL-C reduction (<39 vs ≥39 mg/dL and <30% vs 30%-49% vs ≥50%), study duration (<3 vs ≥3 years), evidence of atherosclerosis (all patients having evidence of atherosclerosis vs most patients not having evidence of atherosclerosis), sample size (<200 vs 200-1000 vs >1000 patients), study design (all patients having stroke or transient ischemic attack vs subgroup of patients having stroke), and coronary artery disease (all patients having concomitant coronary artery disease vs all patients not having concomitant coronary artery disease vs some patients having concomitant coronary artery disease).

The trim-and-fill method to identify and correct for funnel plot asymmetry arising from publication bias was used with Stata/SE, version 15.1 (StataCorp LLC).^16^ To identify any study that might have exerted a disproportionate influence on the summary treatment effect, we removed each individual trial from the meta-analysis 1 at a time. The definition of index strokes varied across studies, and, while all included patients had strokes, it is unclear whether they were all ischemic strokes or whether some may have been hemorrhagic strokes. We therefore conducted a sensitivity test by restricting analysis within patients with ischemic stroke as an entry event. An additional sensitivity test was conducted by excluding trials with participants in the control group not taking statins because the current American College of Cardiology/American Heart Association (ACC/AHA) guidelines suggested that history of ischemic stroke should be regarded as a very high risk of future atherosclerotic cardiovascular disease and statin therapy should be used.^17^ The Cochrane Collaboration’s Review Manager Software Package (RevMan, version 5.4) was used for this meta-analysis.

## Results

We identified 37 full articles for detailed assessment, of which 26 did not meet the inclusion criteria; therefore, the final analysis included 11 randomized clinical trials (eFigure 1 in the Supplement).^18,19,20,21,22,23,24,25,26,27,28^ The characteristics of the included trials are shown in Table 1.^18,19,20,21,22,23,24,25,26,27,28,29,30,31,32^ Overall, 20 163 patients (13 518 men [67.0%]; mean [SD] age, 64.9 [3.7] years) with stroke were enrolled. The mean duration of follow-up was 4 years (range, 1-6.1 years). The final mean LDL-C level, weighted for trial size, was 79 mg/dL in the groups that received more intensive LDL-C lowering and 119 mg/dL in the groups that received less intensive LDL-C lowering. Among the 11 included trials, 6 compared statins vs no statins,^18,19,20,21,22,23^ 3 compared more statins or ezetimibe vs less statins or ezetimibe,^24,25,26^ and 2 compared PCSK9 inhibitors plus statins vs placebo plus statins.^27,28^ Among the 3 trials that compared more statins or ezetimibe vs less statins or ezetimibe, 1 compared ezetimibe plus simvastatin with placebo plus simvastatin,^24^ 1 compared intensive lipid lowering with statin-based therapies with guideline lipid lowering with statin-based therapies,^25^ and 1 compared lower-target (LDL-C level <70 mg/dL) with higher-target (LDL-C level, 90-110 mg/dL) groups.^26^ In the Treat Stroke to Target (TST) trial, 99% of patients in the lower-target group vs 79% in the higher-target group received moderate- or high-intensity statins, and 41% of patients in the lower-target group vs 7% in the higher-target group received combined statins plus ezetimibe.^26^

**Table 1.** Characteristics of Included Trials

Study, publication countries	Population	Intervention treatment, daily dose (except PCSK9 inhibitors)	Comparative treatment, daily dose	Time interval from stroke to randomization	Sample size (women)	Age, y	Study duration, y	LDL-C at baseline, mg/dL	LDL-C difference between the 2 groups after treatment, mg/dL
**Statins vs no statins**									
CARE,^18^ 1999; US and Canada	Subgroup of patients with history of stroke and myocardial infarction	Pravastatin, 40 mg	Placebo	NA	122 (NA)	NA (59 in whole trial)	5	NA (139 in whole trial)	NA (32 mg/dL or 23% in whole trial)
LIPID,^19^ 2000; Australia and New Zealand	Subgroup of patients with history of stroke and myocardial infarction or unstable angina	Pravastatin, 40 mg	Placebo	NA	369 (NA)	NA (62 in whole trial)	6	NA (150 in whole trial)	NA (27 mg/dL or 18% in whole trial)
HPS,^20^ 2004; UK	Subgroup of patients with history of cerebrovascular disease	Simvastatin, 40 mg	Placebo	Stroke within 6 mo	3280 (25%)	66	4.8	131	37 (28%)
SPARCL,^21,29,30,31,32^ 2006, 2008, 2009, 2011; European countries and US	Noncardioembolic stroke or TIA	Atorvastatin, 80 mg	Placebo	1-6 mo	4731 (40%)	63	4.9	133	56 (42%)
Yakusevich et al,^22^ 2012; Russia	First acute ischemic stroke	Simvastatin, 40 mg, plus standard stroke therapy	Standard stroke therapy	24-48 h	183 (56%)	66	1	85	17 (20%)
J-STARS,^23^ 2015; Japan	Noncardioembolic ischemic stroke	Pravastatin, 10 mg	No statin	1 mo-3 y	1578 (31%)	66	4.9	130	21 (16%)
**More statins or ezetimibe vs less statins or ezetimibe**									
IMPROVE-IT,^24^ 2017; European countries, US, Canada, and other countries	Subgroup of patients with history of stroke and acute coronary syndrome within preceding 10 d	Ezetimibe, 10 mg, plus simvastatin, 40 mg	Placebo plus simvastatin, 40 mg	NA	682 (29%)	68	6	88	17 (19%)
PODCAST,^25^ 2017; UK	Patients with ischemic stroke	Intensive lipid lowering	Guideline lipid lowering	Ischemic stroke within previous 3-7 mo	77 (21%)	74	2	78	17 (22%)
TST,^26^ 2020; France and South Korea	Patients with ischemic stroke or TIA and atherosclerotic disease	Lower target (LDL-C, <70 mg/dL); high-intensity statin: 24%; moderate-intensity statin: 76%; ezetimibe: 41%	Higher target (LDL-C, 90-110 mg/dL); high-intensity statin: 9%; moderate-intensity statin: 71% ezetimibe: 7%	Ischemic stroke within past 3 mo or TIA within previous 15 d	2860 (32%)	67	3.5	135	31 (23%)
**PCSK9 inhibitors plus statins vs placebo plus statins**									
ODYSSEY OUTCOMES,^27^ 2019; European countries, US, and New Zealand	Subgroup of patients with history of stroke and acute coronary syndrome 1-12 mo before randomization	Alirocumab, 150 mg, every 2 wk plus statins (atorvastatin,40-80 mg, once daily; rosuvastatin, 20-40 mg, once daily unless not tolerated)	Placebo every 2 wk plus statins (atorvastatin, 40-80 mg, once daily; rosuvastatin, 20-40 mg, once daily unless not tolerated)	NA	944 (32%)	63	2.8	91	NA (53 mg/dL or 58% in whole trial)
FOURIER,^28^ 2020; US, Australia, European countries, and other countries	Subgroup of patients with history of ischemic stroke and additional risk factors	Evolocumab, 140 mg, every 2 wk or 420 mg, every 4 wk plus statins (at least atorvastatin, 20 mg, daily or its equivalent, with or without ezetimibe)	Placebo every 2 wk or every 4 wk plus statins (at least atorvastatin, 20 mg, daily or its equivalent, with or without ezetimibe)	3.3 y	5337 (26%)	65	2.1	93	52 (56%)

Abbreviations: CARE, The Cholesterol and Recurrent Events Study; FOURIER, Further Cardiovascular Outcomes Research With PCSK9 Inhibition in Patients With Elevated Risk; HPS, Heart Protection Study; IMPROVE-IT, Improved Reduction of Outcomes: Vytorin Efficacy International Trial; J-STARS, Japan Statin Treatment Against Recurrent Stroke; LDL-C, low-density lipoprotein cholesterol; LIPID, Long-term Intervention with Pravastatin in Ischaemic Disease; NA, not available; ODYSSEY OUTCOMES, Evaluation of Cardiovascular Outcomes After an Acute Coronary Syndrome During Treatment With Alirocumab; PCSK9, proprotein convertase subtilisin/kexin type 9; PODCAST, Prevention of Decline in Cognition after Stroke Trial; SPARCL, Stroke Prevention by Aggressive Reduction in Cholesterol Levels; TIA, transient ischemic attack; TST, Treat Stroke to Target.

SI conversion factor: To convert LDL-C to millimoles per liter, multiply by 0.0259.

The Cochrane risk-of-bias assessment for the included trials is summarized in eFigure 2 in the Supplement. Four trials had performance bias owing to nonblinding of the intervention.^22,23,25,26^

### Recurrent Stroke

**Figure 1.**
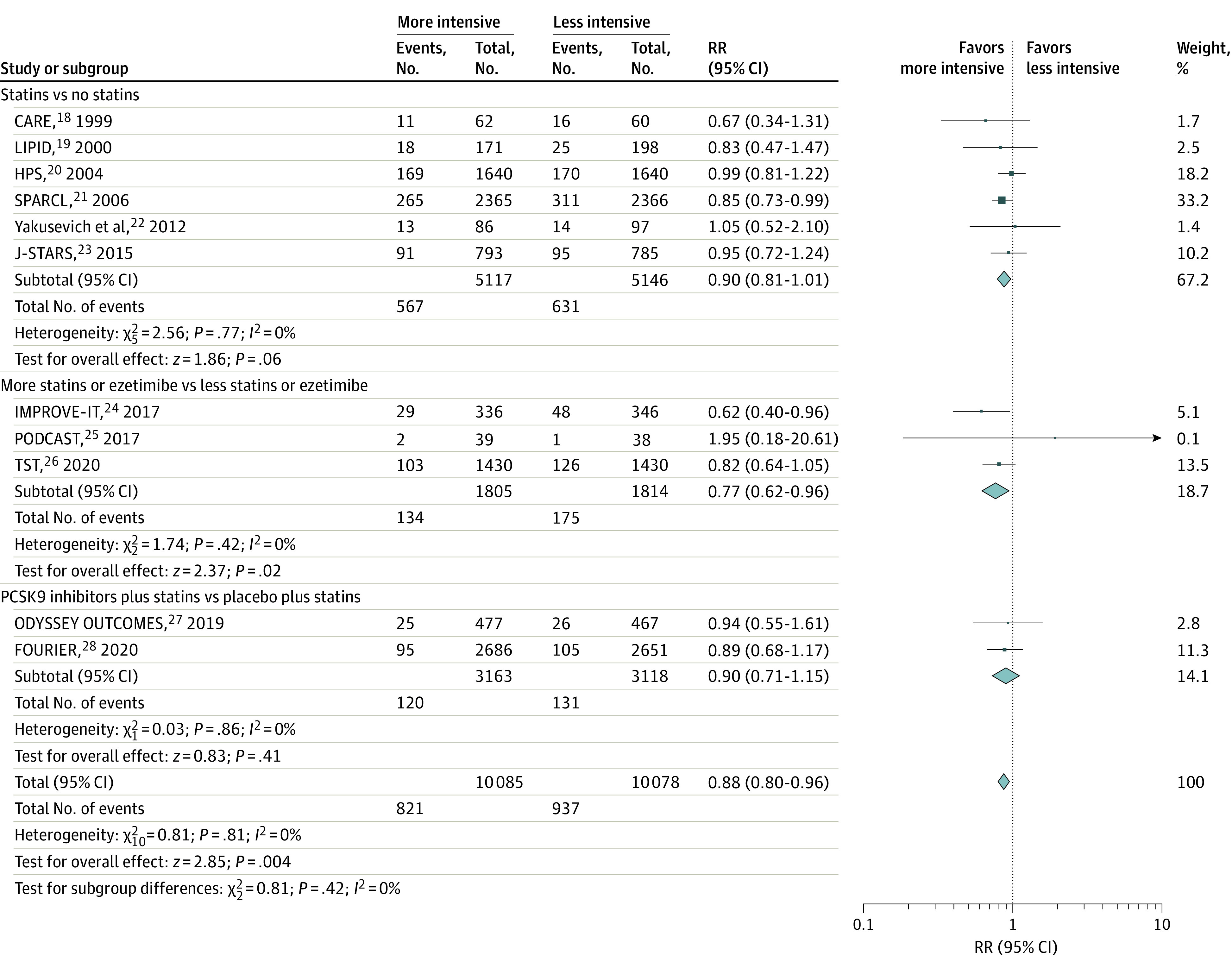
Risk of Recurrent Stroke Relative risk (RR) of recurrent stroke with more intensive vs less intensive low-density lipoprotein cholesterol–lowering statin-based therapies among patients with stroke. Different sizes of markers indicate the different weights used for pooled analysis. CARE indicates the Cholesterol and Recurrent Events Study; FOURIER, Further Cardiovascular Outcomes Research With PCSK9 Inhibition in Patients With Elevated Risk; HPS, Heart Protection Study; IMPROVE-IT, Improved Reduction of Outcomes: Vytorin Efficacy International Trial; J-STARS, Japan Statin Treatment Against Recurrent Stroke; LIPID, Long-term Intervention with Pravastatin in Ischaemic Disease; ODYSSEY OUTCOMES, Evaluation of Cardiovascular Outcomes After an Acute Coronary Syndrome During Treatment With Alirocumab; PCSK9, proprotein convertase subtilisin/kexin type 9; PODCAST, Prevention of Decline in Cognition after Stroke Trial; SPARCL, Stroke Prevention by Aggressive Reduction in Cholesterol Levels; and TST, Treat Stroke to Target.

Pooled results from the fixed-effects model of the 11 included trials showed that more intensive compared with less intensive LDL-C–lowering statin-based therapies were associated with a reduced risk of recurrent stroke (absolute risk, 8.1% vs 9.3%; RR, 0.88; 95% CI, 0.80-0.96; *P* = .004; *I*^2^ = 0%; number needed to treat in 4 years, 90).^18,19,20,21,22,23,24,25,26,27,28^ With respect to the type of intervention, the benefit was not statistically different among the LDL-C–lowering strategies (statins vs no statins: RR, 0.90; 95% CI, 0.81-1.01; more statins or ezetimibe vs less statins or ezetimibe: RR, 0.77; 95% CI, 0.62-0.96; and PCSK9 inhibitors plus statins vs placebo plus statins: RR, 0.90; 95% CI, 0.71-1.15; *P* = .42 for interaction; *I*^2^ = 0%) (Figure 1). Pooled results with the random-effects model obtained similar results.

### MACE, Recurrent Ischemic Stroke, and Myocardial Infarction

Pooled results from 8 trials showed that more intensive compared with less intensive LDL-C–lowering statin-based therapies were associated with a reduced risk of MACE (absolute risk, 13.9% vs 16.7%; RR, 0.83; 95% CI, 0.78-0.89; *P* < .001; *I*^2^ = 0%; number needed to treat, 35) and that the benefit was not statistically different among the LDL-C–lowering strategies (statins vs no statins: RR, 0.83; 95% CI, 0.77-0.90; more statins or ezetimibe vs less statins or ezetimibe: RR, 0.80; 95% CI, 0.68-0.94; and PCSK9 inhibitors plus statins vs placebo plus statins: RR, 0.89; 95% CI, 0.74-1.07; *P* = .68 for interaction; *I*^2^ = 0%) (eFigure 3 in the Supplement).^20,21,22,23,24,25,26,28^ Pooled results from these 8 trials showed that more intensive compared with less intensive LDL-C–lowering statin-based therapies were associated with a reduced risk of recurrent ischemic stroke (absolute risk, 6.3% vs 7.7%; RR, 0.82; 95% CI, 0.74-0.91; *P* < .001; *I*^2^ = 0%; number needed to treat, 72) and that the benefit was not statistically different among the LDL-C–lowering strategies (statins vs no statins: RR, 0.83; 95% CI, 0.73-0.94; more statins or ezetimibe vs less statins or ezetimibe: RR, 0.73; 95% CI, 0.58-0.93; and PCSK9 inhibitors plus statins vs placebo plus statins: RR, 0.92; 95% CI, 0.68-1.24; *P* = .48 for interaction; *I*^2^ = 0%) (eFigure 4 in the Supplement).^20,21,22,23,24,25,26,28^ Pooled results from 7 trials showed that more intensive compared with less intensive LDL-C–lowering statin-based therapies were associated with a reduced risk of myocardial infarction (absolute risk, 3.3% vs 4.3%; RR, 0.73; 95% CI, 0.62-0.86; *P* < .001; *I*^2^ = 0%; number needed to treat, 86) and that the benefit was not statistically different among the LDL-C–lowering strategies (statins vs no statins: RR, 0.67; 95% CI, 0.52-0.87; more statins or ezetimibe vs less statins or ezetimibe: RR, 0.81; 95% CI, 0.60-1.08; and PCSK9 inhibitors plus statins vs placebo plus statins: RR, 0.74; 95% CI, 0.55-0.99; *P* = .65 for interaction; *I*^2^ = 0%) (eFigure 5 in the Supplement).^21,22,23,24,25,26,28^

### Hemorrhagic Stroke

**Figure 2.**
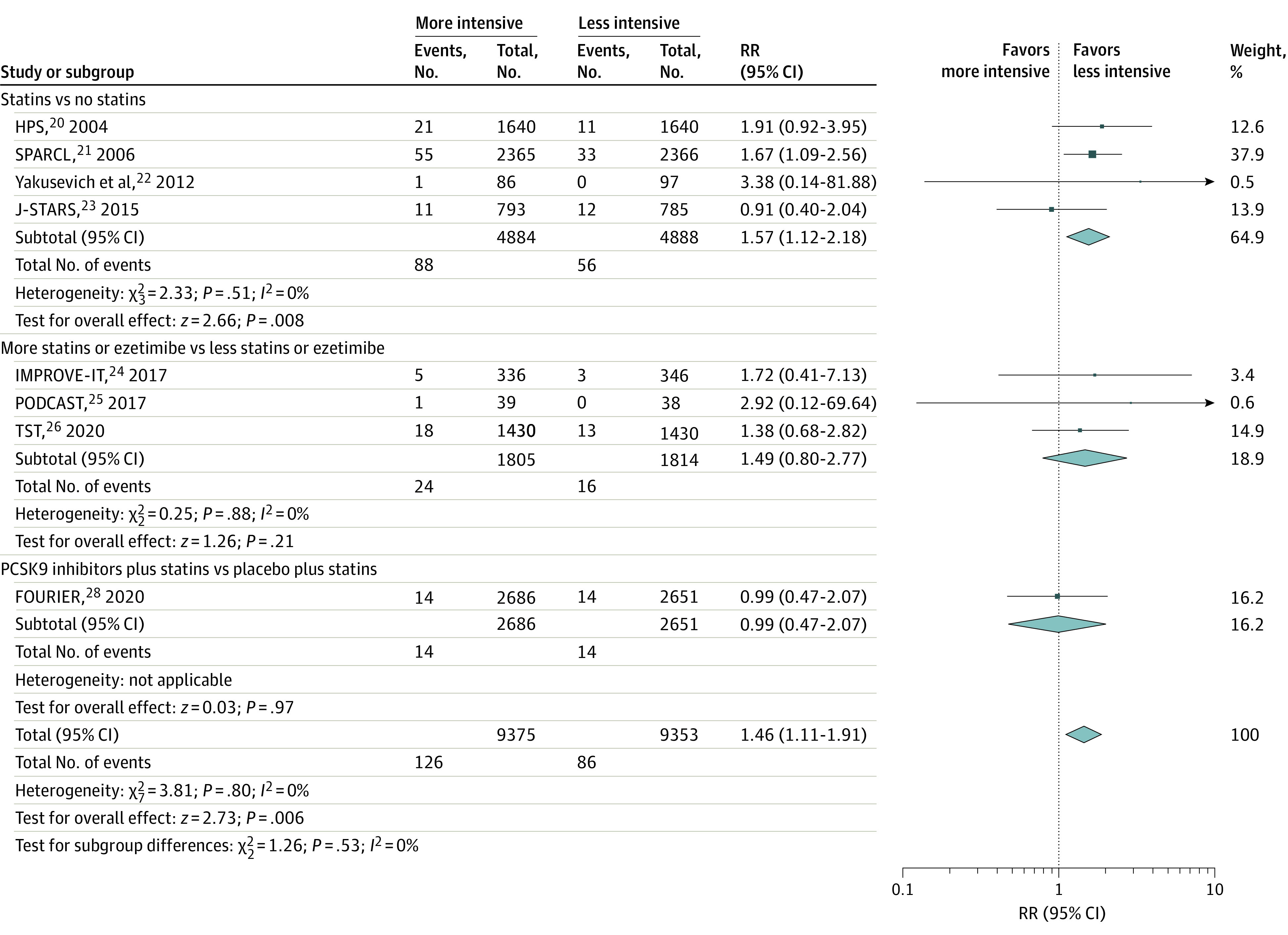
Risk of Hemorrhagic Stroke Relative risk (RR) of hemorrhagic stroke with more intensive vs less intensive low-density lipoprotein cholesterol–lowering statin-based therapies among patients with stroke. Different sizes of markers indicate the different weights used for pooled analysis. FOURIER, Further Cardiovascular Outcomes Research With PCSK9 Inhibition in Patients With Elevated Risk; HPS, Heart Protection Study; IMPROVE-IT, Improved Reduction of Outcomes: Vytorin Efficacy International Trial; J-STARS, Japan Statin Treatment Against Recurrent Stroke; PCSK9, proprotein convertase subtilisin/kexin type 9; PODCAST, Prevention of Decline in Cognition after Stroke Trial; SPARCL, Stroke Prevention by Aggressive Reduction in Cholesterol Levels; and TST, Treat Stroke to Target.

Pooled results from 8 trials showed that more intensive vs less intensive LDL-C–lowering statin-based therapies were associated with an increase in hemorrhagic stroke (RR, 1.46; 95% CI, 1.11-1.91; *P* = .006; *I*^2^ = 0%; number needed to harm, 242).^20,21,22,23,24,25,26,28^ Although point estimates of hemorrhagic stroke were different among the LDL-C–lowering strategies, formal analysis did not show a statistical difference (statins vs no statins: RR, 1.57; 95% CI, 1.12-2.18; more statins or ezetimibe vs less statins or ezetimibe: RR, 1.49; 95% CI, 0.80-2.77; and PCSK9 inhibitors plus statins vs placebo plus statins: RR, 0.99; 95% CI, 0.47-2.07; *P* = .53 for interaction; *I*^2^ = 0%) (Figure 2).

### All-Cause Mortality and Cardiovascular Mortality

Pooled results from 5 trials showed that more intensive vs less intensive LDL-C–lowering statin-based therapies had similar associations with all-cause mortality (RR, 1.02; 95% CI, 0.90-1.15; *P* = .81; *I*^2^ = 0%) (eFigure 6 in the Supplement)^21,22,23,24,26^ and cardiovascular mortality (RR, 0.92; 95% CI, 0.77-1.10; *P* = .37; *I*^2^ = 7%) (eFigure 7 in the Supplement).^21,22,24,26,28^

### New-Onset Diabetes

Pooled results from 3 trials showed that more intensive vs less intensive LDL-C–lowering statin-based therapies were associated with an increase in new-onset diabetes (RR, 1.26; 95% CI, 1.09-1.46; *P* = .002; *I*^2^ = 34%; number needed to harm = 57).^26,28,29^ The risk was not statistically different among the LDL-C–lowering strategies (statins vs no statins: RR, 1.44; 95% CI, 1.14-1.81; more statins or ezetimibe vs less statins or ezetimibe: RR, 1.27; 95% CI, 0.96-1.68; and PCSK9 inhibitors plus statins vs placebo plus statins: RR, 1.06; 95% CI, 0.82-1.37; *P* = .22 for interaction; *I*^2^ = 34%) (eFigure 8 in the Supplement).

### Cognitive Adverse Events

Pooled results from 2 trials showed that more intensive vs less intensive LDL-C–lowering statin-based therapies had similar associations with cognitive adverse events (RR, 0.99; 95% CI, 0.74-1.33; *P* = .94; *I*^2^ = 0%) (eFigure 9 in the Supplement).^23,28^

### Sensitivity Tests

Sensitivity tests excluding individual trials yielded pooled results similar to the overall pooled estimates of the primary outcome. Sensitivity tests conducted by restricting analysis within patients with ischemic stroke as an entry event showed that more intensive LDL-C–lowering statin-based therapies were associated with a reduced risk of recurrent stroke^22,23,25,28,30^ (eFigure 10 in the Supplement), MACE,^22,23,25,26,28,30^ and recurrent ischemic stroke^22,23,25,28,31^ and with a nonsignificant increased risk of hemorrhagic stroke compared with less intensive LDL-C–lowering statin-based therapies.^22,23,25,28,31^ Also, sensitivity tests excluding trials with patients in the control group not taking statins yielded pooled results from trials with more statins or ezetimibe vs less statins or ezetimibe and PCSK9 inhibitors plus statins vs placebo plus statins and showed that more intensive LDL-C–lowering statin-based therapies were associated with a reduced risk of recurrent stroke (eFigure 11 in the Supplement), MACE, recurrent ischemic stroke, and myocardial infarction and with a nonsignificant increased risk of hemorrhagic stroke and new-onset diabetes compared with less intensive LDL-C–lowering statin-based therapies. The association of more intensive vs less intensive LDL-C–lowering statin-based therapies with primary and secondary outcomes among patients with a history of stroke is presented in Table 2.^18,19,20,21,22,23,24,25,26,27,28,29,30,31^

**Table 2.** Association of More Intensive vs Less Intensive LDL-C–Lowering Statin-Based Therapies With Primary and Secondary Outcomes Among Patients With a History of Stroke

End point	LDL-C lowering, No./total No. (%)		RR (95% CI)	NNT or NNH in 4 y (95% CI)
	More intensive	Less intensive		
All eligible trials				
Stroke^18,19,20,21,22,23,24,25,26,27,28^	821/10 085 (8.1)	937/10 078 (9.3)	0.88 (0.80-0.96)	90 (53-269)
MACE^20,21,22,23,24,25,26,28^	1299/9375 (13.9)	1559/9353 (16.7)	0.83 (0.78-0.89)	35 (27-53)
Ischemic stroke^20,21,22,23,24,25,26,28^	586/9375 (6.3)	717/9353 (7.7)	0.82 (0.74-0.91)	72 (50-144)
Hemorrhagic stroke^20,21,22,23,24,25,26,28^	126/9375 (1.3)	86/9353 (0.9)	1.46 (1.11-1.91)	242 (122-1110)
Myocardial infarction^21,22,23,24,25,26,28^	239/7735 (3.1)	329/7713 (4.3)	0.73 (0.62-0.86)	86 (61-166)
All-cause mortality^21,22,23,24,26^	443/5010 (8.8)	440/5024 (8.8)	1.02 (0.90-1.15)	NA
Cardiovascular death^21,22,24,26,28^	224/6903 (3.2)	245/6890 (3.6)	0.92 (0.77-1.10)	NA
New-onset diabetes^26,28,29^	383/4490 (8.5)	303/4479 (6.8)	1.26 (1.09-1.46)	57 (32-163)
Cognitive adverse events^23,28^	86/3749 (2.3)	86/3436 (2.5)	0.99 (0.74-1.33)	NA
Analysis restricted to patients with ischemic stroke as entry event				
Stroke^22,23,25,28,30^	342/4679 (7.3)	395/4654 (8.5)	0.87 (0.76-0.99)	90 (49-1176)
MACE^22,23,25,26,28,30^	628/5899 (10.6)	771/5883 (13.1)	0.82 (0.74-0.90)	42 (29-76)
Ischemic stroke^22,23,25,28,31^	277/4708 (5.9)	334/4673 (7.1)	0.83 (0.71-0.96)	83 (49-352)
Hemorrhagic stroke^22,23,25,28,31^	55/4708 (1.2)	37/4673 (0.8)	1.47 (0.97-2.21)	NA
All-cause mortality^22,23,30^	164/1954 (8.4)	159/1965 (8.1)	1.05 (0.85-1.29)	NA
Excluding trials with patients in control group not taking statins				
Stroke^24,25,26,27,28^	254/4968 (5.1)	306/4932 (6.2)	0.83 (0.70-0.97)	95 (54-538)
MACE^24,25,26,28^	411/4491 (9.2)	489/4465 (11.0)	0.84 (0.74-0.95)	57 (35-182)
Ischemic stroke^24,25,26,28^	192/4491 (4.3)	240/4465 (5.4)	0.80 (0.66-0.96)	93 (54-463)
Hemorrhagic stroke^24,25,26,28^	38/4491 (0.8)	30/4465 (0.7)	1.26 (0.78-2.02)	NA
Myocardial infarction^24,25,26,28^	147/4491 (3.3)	191/4465 (4.3)	0.77 (0.63-0.95)	101 (63-465)
All-cause mortality^24,26^	171/1766 (9.7)	178/1776 (10.0)	0.98 (0.81-1.18)	NA
Cardiovascular death^24,26,28^	133/4452 (3.0)	131/4427 (3.0)	1.01 (0.75-1.35)	NA
New-onset diabetes^26,28^	217/2585 (8.4)	188/2581 (7.3)	1.15 (0.95-1.39)	NA

Abbreviations: LDL-C, low-density lipoprotein cholesterol; MACE, major adverse cardiovascular event; NA, not applicable; NNH, number needed to harm; NNT, number needed to treat; RR, risk ratio.

### Metaregression

Metaregression did not demonstrate a linear association between degree of LDL-C lowering and recurrent stroke rate (eFigure 12 in the Supplement).

### Subgroup Analysis

**Figure 3.**
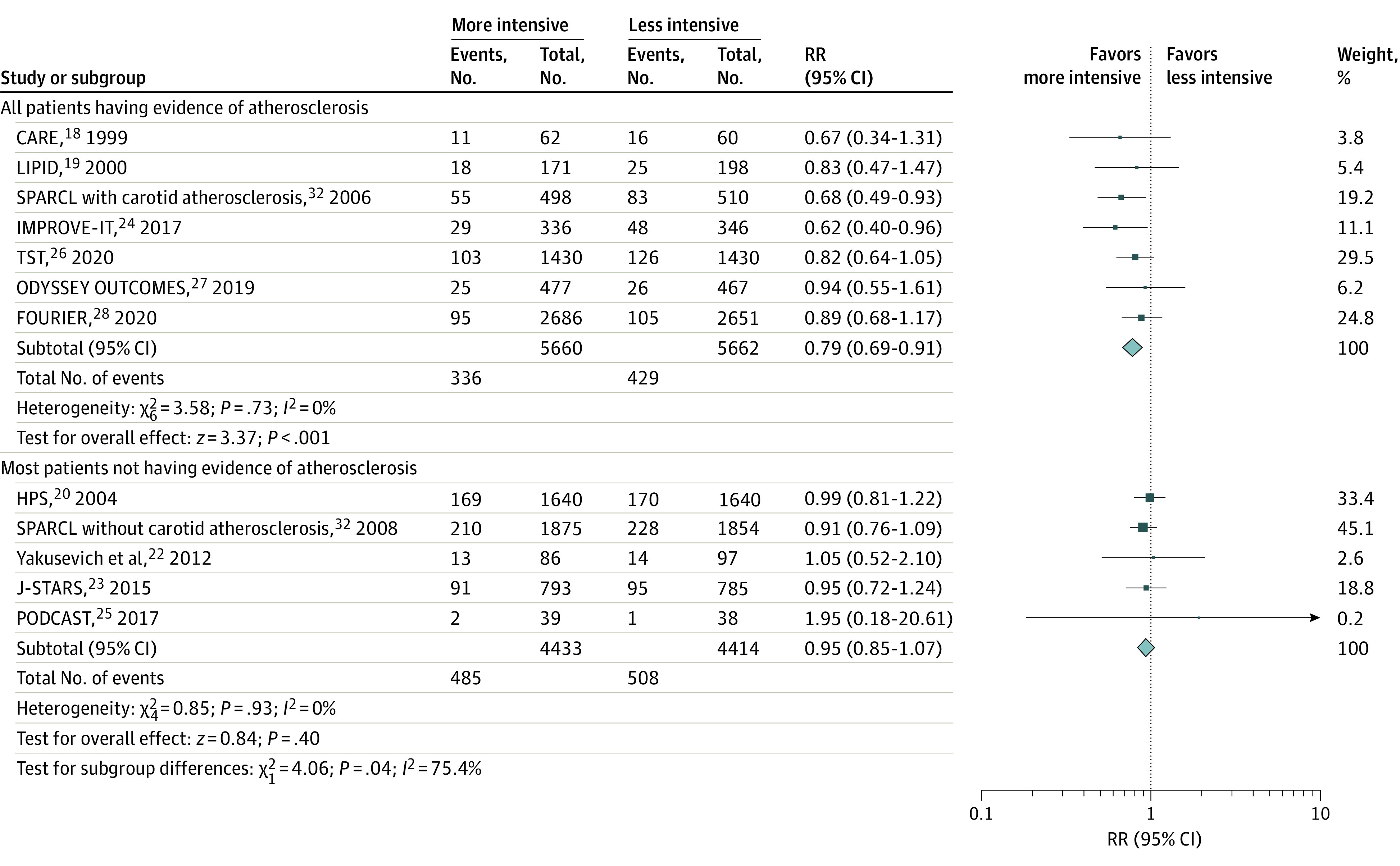
Evidence of Atherosclerosis Relative risk (RR) with 95% CI of recurrent stroke with more intensive vs less intensive low-density lipoprotein cholesterol–lowering statin-based therapies among patients with stroke having or not having evidence of atherosclerosis. Different sizes of markers indicate the different weights used for pooled analysis. CARE indicates the Cholesterol and Recurrent Events Study; FOURIER, Further Cardiovascular Outcomes Research With PCSK9 Inhibition in Patients With Elevated Risk; HPS, Heart Protection Study; IMPROVE-IT, Improved Reduction of Outcomes: Vytorin Efficacy International Trial; J-STARS, Japan Statin Treatment Against Recurrent Stroke; LIPID, Long-term Intervention with Pravastatin in Ischaemic Disease; ODYSSEY OUTCOMES, Evaluation of Cardiovascular Outcomes After an Acute Coronary Syndrome During Treatment With Alirocumab; PODCAST, Prevention of Decline in Cognition after Stroke Trial; SPARCL, Stroke Prevention by Aggressive Reduction in Cholesterol Levels; and TST, Treat Stroke to Target.

More intensive vs less intensive LDL-C–lowering statin-based therapies were associated with a reduced risk of recurrent stroke in trials with all patients having evidence of atherosclerosis (RR, 0.79; 95% CI, 0.69-0.91)^18,19,24,26,27,28,32^ but not in trials with most patients not having evidence of atherosclerosis (RR, 0.95; 95% CI, 0.85-1.07; *P* = .04 for interaction; *I*^2^ = 75%) (Figure 3).^20,22,23,25,32^ Otherwise, no obvious heterogeneity was found in other subgroup analyses (eFigure 13 in the Supplement).

### Publication Bias

There was no obvious publication bias assessed by the trim-and-fill method for the primary outcome (eFigure 14 in the Supplement).

## Discussion

The present meta-analysis, comprising 11 randomized clinical trials with 20 163 individuals with a history of stroke, revealed that more intensive LDL-C–lowering statin-based therapies were associated with a 12% reduced risk of recurrent stroke and a 17% reduced risk of MACE, as well as a 46% increased risk of hemorrhagic stroke, compared with less intensive LDL-C–lowering statin-based therapies. In more practical terms, the number needed to treat to prevent a stroke in 4 years was 90, and the number needed to prevent a MACE was 35, whereas the number needed to harm was 242 for a hemorrhagic stroke. Also, more intensive LDL-C–lowering statin-based therapies were associated with a reduced risk of recurrent ischemic stroke and myocardial infarction, but were associated with a higher risk for new-onset diabetes, compared with less intensive LDL-C–lowering statin-based therapies.

Although the latest ACC/AHA cholesterol practice guidelines suggest that hemorrhagic stroke is not a statin-associated adverse effect,^17^ our meta-analysis found that such a risk exists for patients with stroke; this finding is consistent with that noted in a recently published meta-analysis.^8^ We found that the risk of hemorrhagic stroke became statistically insignificant and that the effect size was smaller when we excluded trials with patients in the control group who were not taking statins. Evolocumab plus statins compared with placebo plus statins reduced the LDL-C level by 52 mg/dL, or 56%, but did not increase the risk of hemorrhagic stroke among patients with a history of ischemic stroke.^28^ Post hoc analysis of the Improved Reduction of Outcomes: Vytorin Efficacy International Trial (IMPROVE-IT) showed that the risk of hemorrhagic stroke was not increased among patients with an LDL-C level lower than 30 mg/dL compared with patients with an LDL-C level of higher than 70 mg/dL.^33^ Taken together, the risk of hemorrhagic stroke might not be associated with LDL-C levels or the magnitude of LDL-C–lowering therapies, but it might be associated with the antithrombotic properties possessed by statins that alter both coagulation and platelet activation.^7^

A prior meta-analysis suggested that a reduction of MACE is proportional to the magnitude of the LDL-C lowering statin-based therapies in secondary prevention for patients with established atherosclerotic cardiovascular disease,^3^ but such a finding was not confirmed by the metaregression performed in our study. The heterogeneity of causes of index stroke, as well as recurrent stroke, may be 1 major factor associated with such a phenomenon. The benefits associated with LDL-C–lowering statin-based therapies vary among patients with stroke owing to the different causes, and there are concerns that such a strategy may not be universally beneficial to all patients with ischemic stroke.^34^ We found that more intensive LDL-C–lowering statin-based therapies were associated with a reduced risk of recurrent stroke only in trials with all patients having evidence of atherosclerosis. On the other hand, patients with ischemic stroke who do not show evidence of atherosclerosis may not experience reduction in the risk of recurrent stroke but may expose themselves to an unnecessary increased risk of hemorrhagic stroke and new-onset diabetes when intensive LDL-C–lowering statin-based therapies are applied.

The recently issued 2021 AHA/American Stroke Association guideline for recurrent stroke prevention recommends that, for patients with noncardioembolic ischemic stroke and an LDL-C level of higher than 100 mg/dL, atorvastatin, 80 mg daily, is indicated to reduce recurrent stroke risk.^35^ However, this recommendation was based primarily on results from a single large trial.^21^ Moreover, atorvastatin, 80 mg daily, is not the only efficacious, intensive LDL-C–lowering strategy. For instance, in the lower-target group of the TST Trial, an LDL-C level of 65 mg/dL was achieved in only 24% of patients in this target group receiving high-intensity statins, while a much higher percentage of patients in this group received combined statins plus ezetimibe (41%).^26^ Our meta-analysis of data from several clinical trials suggested that more intensive LDL-C–lowering statin-based therapies were associated with an increased risk of hemorrhagic stroke, a risk possibly exacerbated by use of high-intensity statins,^7,8^ and that there was no reduced risk of recurrent stroke among patients not having evidence of atherosclerosis. Although we agree that LDL-C–lowering statin-based therapies are indicated for patients with ischemic stroke and an LDL-C level of higher than 100 mg/dL, high-intensity statins, such as atorvastatin, 80 mg daily, should probably be used only when there is evidence of atherosclerosis.

The lowest LDL-C level among patients in the included trials was 31 mg/d, as shown in a trial with a PCSK9 inhibitor plus statins; there was a nonsignificant reduction in the risk of recurrent stroke, and the risk of hemorrhagic stroke was not increased.^28^ Another included trial found LDL-C levels of 51 mg/dL among patients who received ezetimibe plus simvastatin vs 68 mg/dL among those who received simvastatin alone; ezetimibe plus simvastatin compared with simvastatin alone was associated with a reduced risk of recurrent stroke and a nonsignificantly increased risk of hemorrhagic stroke.^24^ The TST Trial compared lower-target with higher-target groups and found LDL-C levels of 65 mg/dL in the lower-target group vs 96 mg/dL in the higher-target group; the lower-target group compared with higher-target group was associated with a reduced risk of MACE, as well as a nonsignificant reduction in the risk of recurrent stroke and a nonsignificantly increased risk of hemorrhagic stroke.^26^ Based on these findings, it might be reasonable to lower LDL-C below 70 mg/dL with statin-based therapies for patients with ischemic stroke and evidence of atherosclerosis. However, the lowest level below which it is not recommended to lower LDL-C might not be known based on the evidence currently available.

### Limitations

Our study has several limitations. First, the purpose of several of the included trials was not to primarily evaluate more intensive vs less intensive LDL-C–lowering statin-based therapies for patients with ischemic stroke, and in such studies, we used a subgroup of patients with a history of stroke for this meta-analysis. In such situations, the characteristics of the index stroke and the duration between the index stroke and the trial initiation were usually vague. Second, the sample sizes among the trials varied. Sample sizes were fewer than 200 patients in 3 studies and between 200 and 1000 patients in another 3 studies. Although subgroup analysis did not find an association of sample size with the primary outcome, the disparity in study sizes may still be regarded as a limitation of this meta-analysis. Third, the 11 included trials represented the mostly high-income countries of Europe, North America, Australia, New Zealand, Japan, and South Korea. One included trial performed in Japan comparing pravastatin, 10 mg daily, with placebo did not show a reduction in the risk of recurrent stroke.^23^ In the TST Trial, although the lower-target strategy was superior to the higher-target strategy in the French population, the benefit of the lower target was not shown for either major cardiovascular events or in recurrent stroke when South Korean patients were analyzed separately.^26,34^ Because the risk of recurrent stroke was not reduced by LDL-C–lowering statin-based therapies in randomized clinical trials of Asian populations, it is therefore not known whether the benefit associated with more intensive LDL-C–lowering statin-based therapies for secondary stroke prevention should be generalized to Asian populations.

## Conclusions

This meta-analysis of accumulated clinical trial data suggests that more intensive compared with less intensive LDL-C–lowering statin-based therapies might be associated with a reduced risk of recurrent stroke among patients with ischemic stroke, but this reduced risk might be confined to patients with evidence of atherosclerosis. Also, more intensive compared with less intensive LDL-C–lowering statin-based therapies might be associated with a reduced risk of MACE, ischemic stroke, and myocardial infarction but might also be associated with an increased risk of hemorrhagic stroke and new-onset diabetes. For patients without evidence of atherosclerosis, intensive LDL-C–lowering statin-based therapies might not be needed in most situations considering the uncertain benefits of secondary stroke prevention and the increased risk of hemorrhagic stroke associated with intensive LDL-C lowering. Also, further data from randomized clinical trials are warranted to elucidate whether intensive LDL-C–lowering statin-based therapies is beneficial for certain racial and ethnic groups, such as Asian individuals.

## Notes

Supplement. eFigure 1. Study Selection eFigure 2. Risk of Bias eFigure 3: MACE eFigure 4. Recurrent Ischemic Stroke eFigure 5. Myocardial Infarction eFigure 6. All-Cause Mortality eFigure 7. Cardiovascular Mortality eFigure 8. New-Onset Diabetes eFigure 9. Cognitive Adverse Events eFigure 10. Recurrent Stroke eFigure 11. Recurrent Stroke eFigure 12. Meta-regression eFigure 13. Subgroup Analyses eFigure 14. Publication Bias eReferences.
