# Supplementary material for: Association Between Intensity of Low-Density Lipoprotein Cholesterol Reduction With Statin-Based Therapies and Secondary Stroke Prevention: A Meta-analysis of Randomized Clinical Trials
Source: JAMA Neurol. 2022 Feb 21;79(4):349–58. doi: 10.1001/jamaneurol.2021.5578 (PMC8861901; doi:10.1001/jamaneurol.2021.5578)
Supplement: Supplement. — eFigure 1. Study Selection eFigure 2. Risk of Bias eFigure 3: MACE eFigure 4. Recurrent Ischemic Stroke eFigure 5. Myocardial Infarction eFigure 6. All-Cause Mortality eFigure 7. Cardiovascular Mortality eFigure 8. New-Onset Diabetes eFigure 9. Cognitive Adverse Events eFigure 10. Recurrent Stroke eFigure 11. Recurrent Stroke eFigure 12. Meta-regression eFigure 13. Subgroup Analyses eFigure 14. Publication Bias eReferences. [file jamaneurol-e215578-s001.pdf]

Supplementary Online Content

Lee M, Cheng CY, Wu YL, Lee JD, Hsu CY, Ovbiagele B. Association between intensity of low-density lipoprotein cholesterol reduction with statin-based therapies and secondary stroke prevention: a meta-analysis of randomized clinical trials. *JAMA Neurol.* Published online February 21, 2022. doi:10.1001/jamaneurol.2021.5578

eFigure 1. Study Selection
eFigure 2. Risk of Bias
eFigure 3: MACE
eFigure 4. Recurrent Ischemic Stroke
eFigure 5. Myocardial Infarction
eFigure 6. All-Cause Mortality
eFigure 7. Cardiovascular Mortality
eFigure 8. New-Onset Diabetes
eFigure 9. Cognitive Adverse Events
eFigure 10. Recurrent Stroke
eFigure 11. Recurrent Stroke
eFigure 12. Meta-regression
eFigure 13. Subgroup Analyses
eFigure 14. Publication Bias
eReferences.

This supplementary material has been provided by the authors to give readers additional information about their work.

© 2022 Lee M et al. *JAMA Neurology*.

eFigure 1. Study Selection

Legends: Flow of study selection

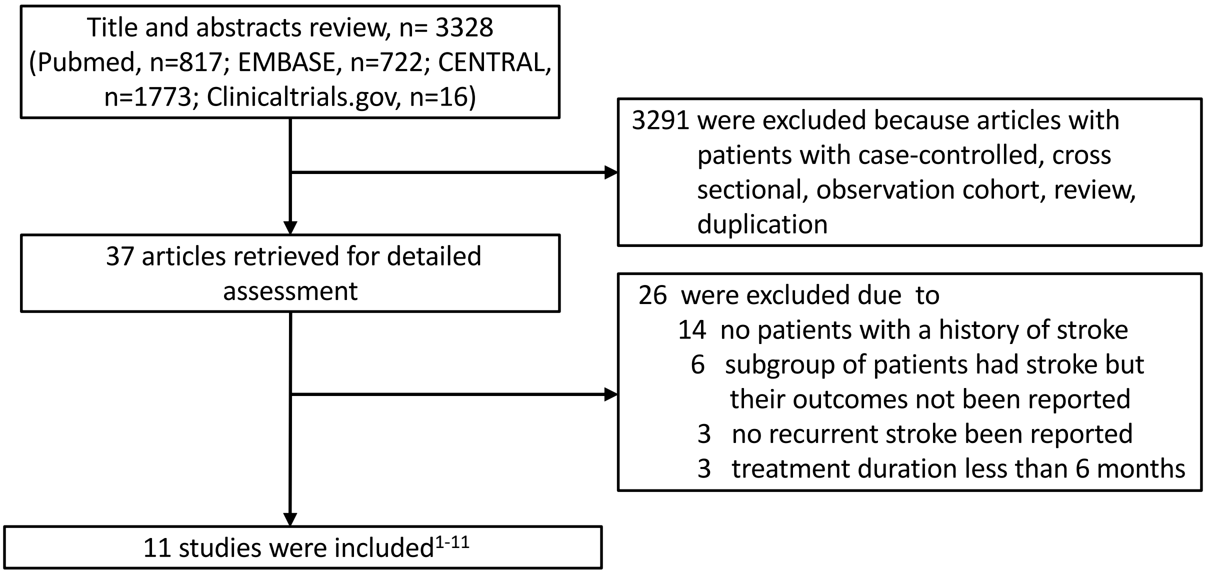

© 2022 Lee M et al. *JAMA Neurology*.

eFigure 2. Risk of Bias

Legends: Risk of bias for included trials

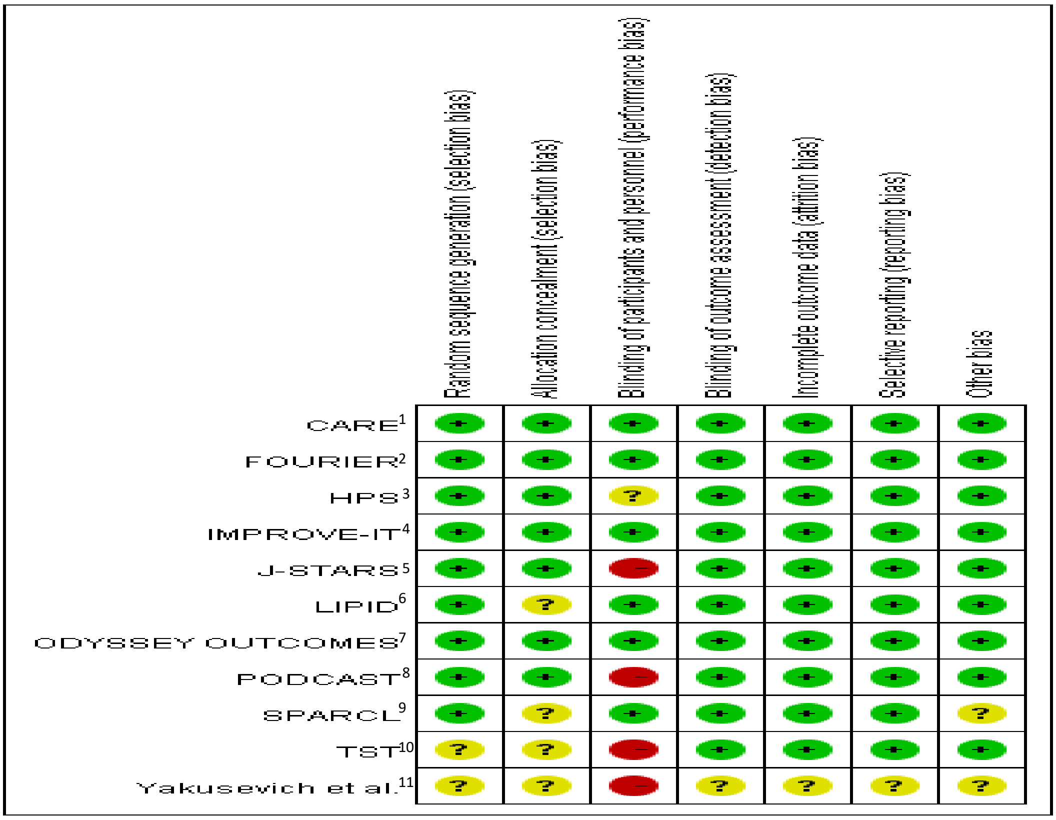

© 2022 Lee M et al. *JAMA Neurology*.

eFigure 3: MACE

Legends: Relative risk with 95% confidence interval of MACE in more intensive vs less intensive LDL-C lowering with statin-based therapies in patients with stroke

MACE: Major adverse cardiovascular events

Study or Subgroup	More-intensive Events	More-intensive Total	Less-intensive Events	Less-intensive Total	Weight	Risk Ratio M-H, Fixed, 95% CI	Year
**3.1.1 Statin vs. No statin**							
HPS [3]	406	1640	488	1640	31.3%	0.83 [0.74, 0.93]	2004
SPARCL[9]	334	2365	407	2366	26.1%	0.82 [0.72, 0.94]	2006
Yakusevich et al.[11]	23	86	28	97	1.7%	0.93 [0.58, 1.48]	2012
J-STARS[5]	125	793	147	785	9.5%	0.84 [0.68, 1.05]	2015
**Subtotal (95% CI)**		**4884**		**4888**	**68.6%**	**0.83 [0.77, 0.90]**	
Total events	888		1070				
Heterogeneity: Chi² = 0.25, df = 3 (P = 0.97); I² = 0%							
Test for overall effect: Z = 4.58 (P < 0.00001)							
**3.1.2 More statin/ezetimibe vs. Less statin/ezetimibe**							
IMPROVE-IT [4]	93	336	117	346	7.4%	0.82 [0.65, 1.03]	2017
PODCAST [8]	3	39	1	38	0.1%	2.92 [0.32, 26.88]	2017
TST [10]	113	1430	147	1430	9.4%	0.77 [0.61, 0.97]	2020
**Subtotal (95% CI)**		**1805**		**1814**	**16.9%**	**0.80 [0.68, 0.94]**	
Total events	209		265				
Heterogeneity: Chi² = 1.46, df = 2 (P = 0.48); I² = 0%							
Test for overall effect: Z = 2.69 (P = 0.007)							
**3.1.3 PCSK9 inhibitor plus statin vs. Placebo plus statin**							
FOURIER[2]	202	2686	224	2651	14.5%	0.89 [0.74, 1.07]	2020
**Subtotal (95% CI)**		**2686**		**2651**	**14.5%**	**0.89 [0.74, 1.07]**	
Total events	202		224				
Heterogeneity: Not applicable							
Test for overall effect: Z = 1.25 (P = 0.21)							
**Total (95% CI)**		**9375**		**9353**	**100.0%**	**0.83 [0.78, 0.89]**	
Total events	1299		1559				
Heterogeneity: Chi² = 2.46, df = 7 (P = 0.93); I² = 0%							
Test for overall effect: Z = 5.35 (P < 0.00001)							
Test for subgroup differences: Chi² = 0.76, df = 2 (P = 0.68), I² = 0%							

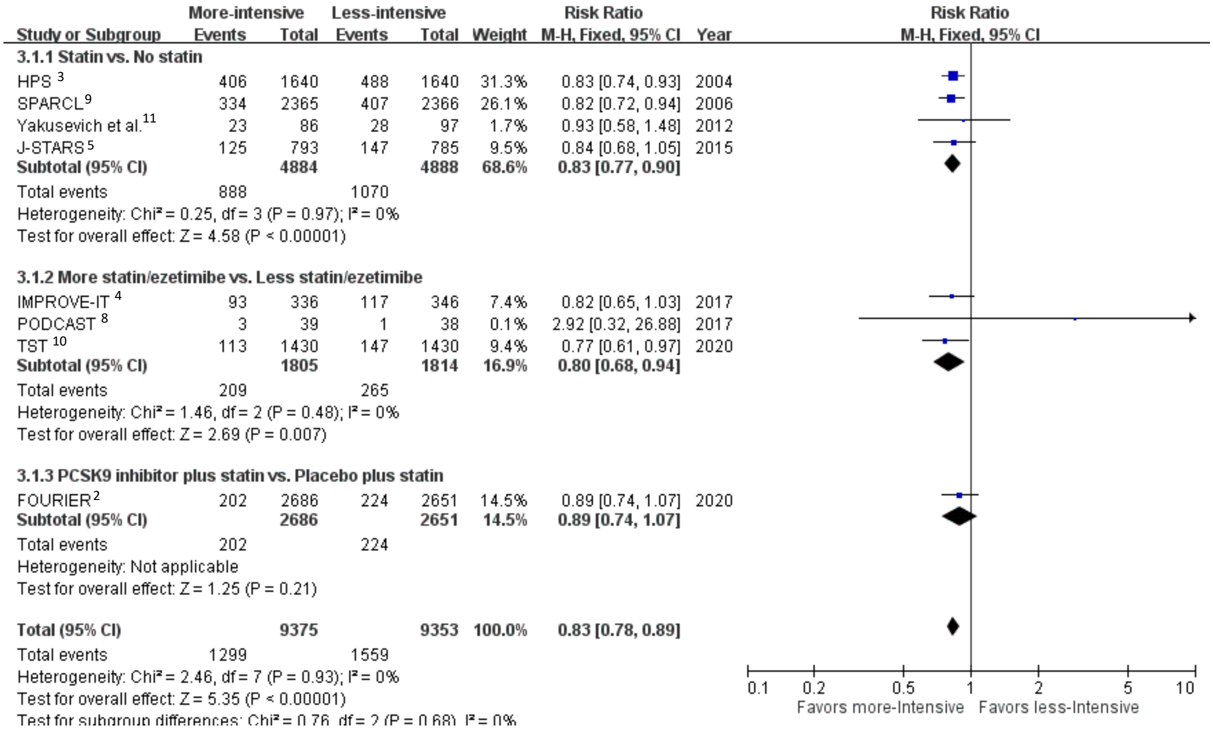

© 2022 Lee M et al. *JAMA Neurology*.

eFigure 4. Recurrent Ischemic Stroke

Legends: Relative risk with 95% confidence interval of recurrent ischemic stroke in more intensive vs less intensive LDL-C lowering with statin-based therapies in patients with stroke

Study or Subgroup	More-intensive Events	More-intensive Total	Less-intensive Events	Less-intensive Total	Weight	Risk Ratio M-H, Fixed, 95% CI	Year
**4.1.1 Statin vs. No statin**							
HPS[3]	100	1640	122	1640	17.0%	0.82 [0.63, 1.06]	2004
SPARCL[9]	218	2365	274	2366	38.2%	0.80 [0.67, 0.94]	2006
Yakusevich et al.[11]	12	86	14	97	1.8%	0.97 [0.47, 1.97]	2012
J-STARS[5]	64	793	67	785	9.4%	0.95 [0.68, 1.31]	2015
**Subtotal (95% CI)**		**4884**		**4888**	**66.5%**	**0.83 [0.73, 0.94]**	
Total events	394		477				
Heterogeneity: Chi² = 1.03, df = 3 (P = 0.79); I² = 0%							
Test for overall effect: Z = 2.91 (P = 0.004)							
**4.1.2 More statin/ezetimibe vs. Less statin/ezebimibe**							
IMPROVE-IT[4]	23	336	44	346	6.1%	0.54 [0.33, 0.87]	2017
PODCAST[8]	1	39	1	38	0.1%	0.97 [0.06, 15.02]	2017
TST[10]	88	1430	109	1430	15.2%	0.81 [0.62, 1.06]	2020
**Subtotal (95% CI)**		**1805**		**1814**	**21.4%**	**0.73 [0.58, 0.93]**	
Total events	112		154				
Heterogeneity: Chi² = 2.11, df = 2 (P = 0.35); I² = 5%							
Test for overall effect: Z = 2.60 (P = 0.009)							
**4.1.3 PCSK9 inhibitor plus statin vs. Placebo plus statin**							
FOURIER[2]	80	2686	86	2651	12.1%	0.92 [0.68, 1.24]	2020
**Subtotal (95% CI)**		**2686**		**2651**	**12.1%**	**0.92 [0.68, 1.24]**	
Total events	80		86				
Heterogeneity: Not applicable							
Test for overall effect: Z = 0.56 (P = 0.58)							
**Total (95% CI)**		**9375**		**9353**	**100.0%**	**0.82 [0.74, 0.91]**	
Total events	586		717				
Heterogeneity: Chi² = 4.56, df = 7 (P = 0.71); I² = 0%							
Test for overall effect: Z = 3.75 (P = 0.0002)							
Test for subgroup differences: Chi² = 1.46, df = 2 (P = 0.48), I² = 0%							

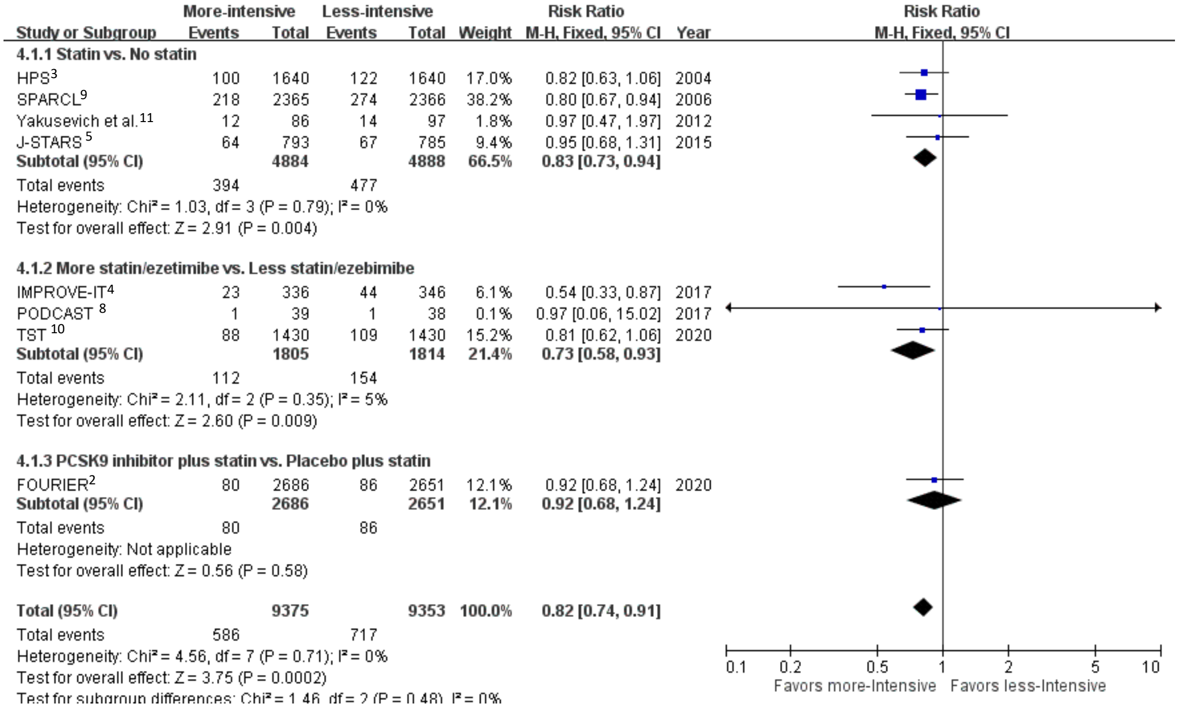

© 2022 Lee M et al. *JAMA Neurology*.

eFigure 5. Myocardial Infarction

Legends: Relative risk with 95% confidence interval of myocardial infarction in more intensive vs less intensive LDL-C lowering with statin-based therapies in patients with stroke

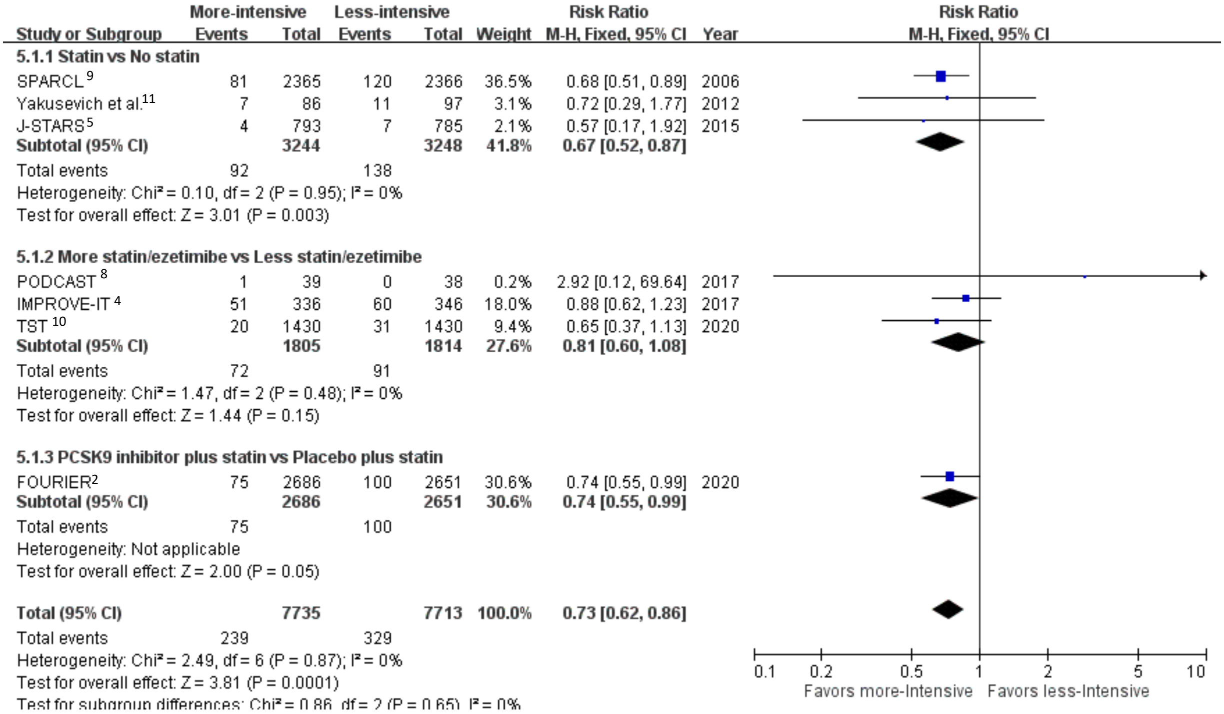

© 2022 Lee M et al. *JAMA Neurology*.

eFigure 6. All-Cause Mortality

Legends: Relative risk with 95% confidence interval of all-cause mortality in more intensive vs less intensive LDL-C lowering with statin-based therapies in patients with stroke

Study or Subgroup	More-intensive Events	More-intensive Total	Less-intensive Events	Less-intensive Total	Weight	Risk Ratio M-H, Fixed, 95% CI	Year
**6.1.1 Statin vs. no statin**							
SPARCL[9]	216	2365	211	2366	48.2%	1.02 [0.85, 1.23]	2006
Yakusevich et al.[11]	13	86	16	97	3.4%	0.92 [0.47, 1.79]	2012
J-STARS[5]	43	793	35	785	8.0%	1.22 [0.79, 1.88]	2015
**Subtotal (95% CI)**		**3244**		**3248**	**59.6%**	**1.04 [0.89, 1.23]**	
Total events	272		262				
Heterogeneity: Chi² = 0.66, df = 2 (P = 0.72); I² = 0%							
Test for overall effect: Z = 0.52 (P = 0.60)							
**6.1.2 More statin/ezetimibe vs Less statin/ezetimibe**							
IMPROVE-IT[4]	83	336	85	346	19.1%	1.01 [0.77, 1.31]	2017
TST[10]	88	1430	93	1430	21.2%	0.95 [0.71, 1.25]	2020
**Subtotal (95% CI)**		**1766**		**1776**	**40.4%**	**0.97 [0.80, 1.18]**	
Total events	171		178				
Heterogeneity: Chi² = 0.10, df = 1 (P = 0.76); I² = 0%							
Test for overall effect: Z = 0.26 (P = 0.79)							
**Total (95% CI)**		**5010**		**5024**	**100.0%**	**1.02 [0.90, 1.15]**	
Total events	443		440				
Heterogeneity: Chi² = 1.00, df = 4 (P = 0.91); I² = 0%							
Test for overall effect: Z = 0.25 (P = 0.81)							
Test for subgroup differences: Chi² = 0.29, df = 1 (P = 0.59), I² = 0%							

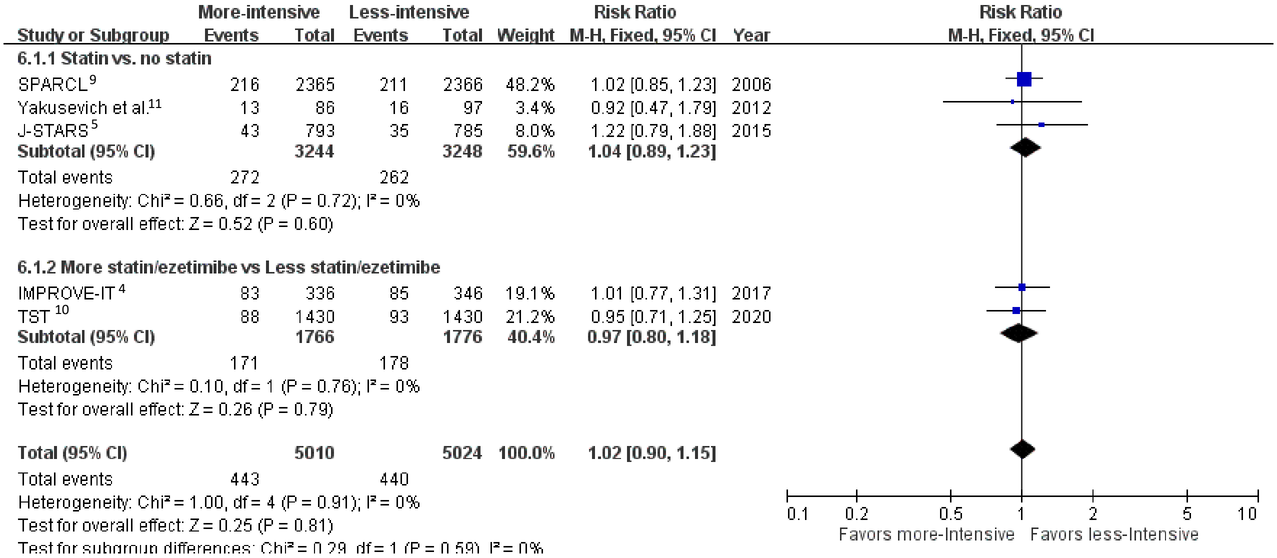

© 2022 Lee M et al. *JAMA Neurology*.

eFigure 7. Cardiovascular Mortality

Legends: Relative risk with 95% confidence interval of cardiovascular mortality in more intensive vs less intensive LDL-C lowering with statin-based therapies in patients with stroke

	More-intensive		Less-intensive			Risk Ratio	
Study or Subgroup	Events	Total	Events	Total	Weight	M-H, Fixed, 95% CI	Year
**7.1.1 Statin vs. No statin**							
SPARCL [9]	78	2365	98	2366	40.2%	0.80 [0.59, 1.07]	2006
Yakusevich et al. [11]	13	86	16	97	6.2%	0.92 [0.47, 1.79]	2012
**Subtotal (95% CI)**		**2451**		**2463**	**46.3%**	**0.81 [0.62, 1.06]**	
Total events	91		114				
Heterogeneity: Chi² = 0.14, df = 1 (P = 0.71); I² = 0%							
Test for overall effect: Z = 1.52 (P = 0.13)							
**7.1.2 More statin/ezetimibe vs. Less statin/ezetimibe**							
IMPROVE-IT [4]	38	336	34	346	13.7%	1.15 [0.74, 1.78]	2017
TST [10]	22	1430	32	1430	13.1%	0.69 [0.40, 1.18]	2020
**Subtotal (95% CI)**		**1766**		**1776**	**26.9%**	**0.92 [0.66, 1.30]**	
Total events	60		66				
Heterogeneity: Chi² = 2.13, df = 1 (P = 0.14); I² = 53%							
Test for overall effect: Z = 0.46 (P = 0.65)							
**7.1.3 PCSK9 inhibitor plus statin vs. Placebo plus statin**							
FOURIER [2]	73	2686	65	2651	26.8%	1.11 [0.80, 1.54]	2020
**Subtotal (95% CI)**		**2686**		**2651**	**26.8%**	**1.11 [0.80, 1.54]**	
Total events	73		65				
Heterogeneity: Not applicable							
Test for overall effect: Z = 0.61 (P = 0.54)							
**Total (95% CI)**		**6903**		**6890**	**100.0%**	**0.92 [0.77, 1.10]**	
Total events	224		245				
Heterogeneity: Chi² = 4.30, df = 4 (P = 0.37); I² = 7%							
Test for overall effect: Z = 0.90 (P = 0.37)							
Test for subgroup differences: Chi² = 2.06, df = 2 (P = 0.36), I² = 2.8%							

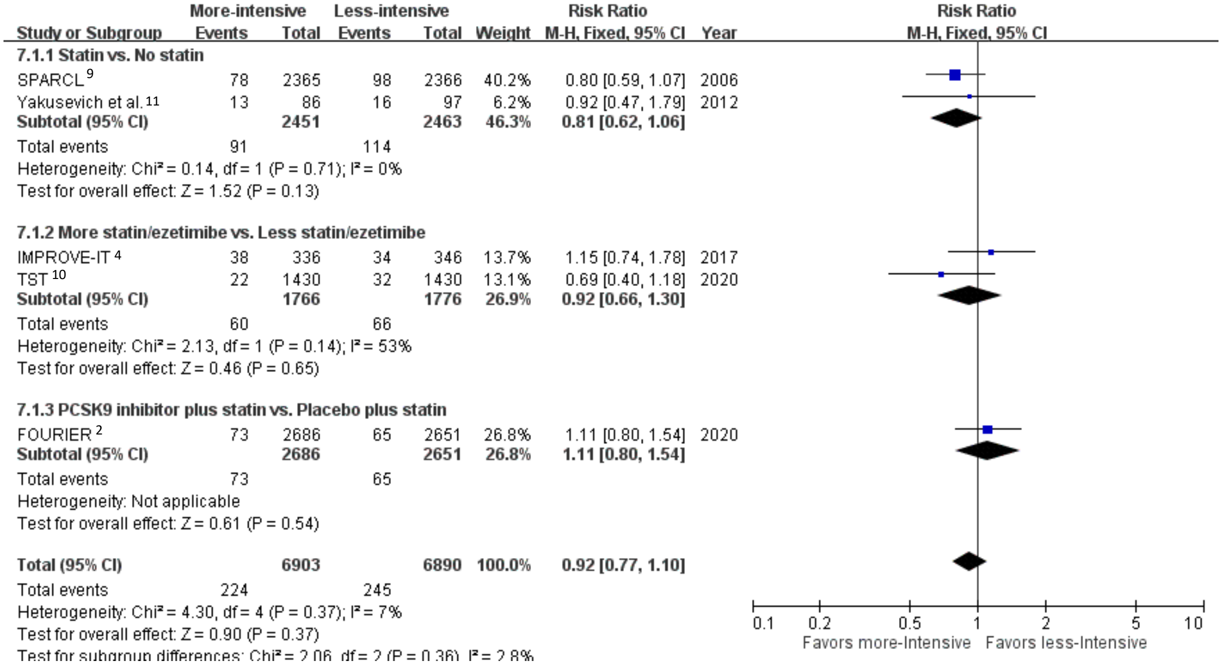

© 2022 Lee M et al. *JAMA Neurology*.

eFigure 8. New-Onset Diabetes

Legends: Relative risk with 95% confidence interval of new-onset diabetes in more intensive vs less intensive LDL-C lowering with statin-based therapies in patients with stroke

	More-intensive		Less-intensive			Risk Ratio	
**Study or Subgroup**	**Events**	**Total**	**Events**	**Total**	**Weight**	**M-H, Fixed, 95% CI**	**Year**
**8.1.1 Stains vs No statins**							
SPARCL [12]	166	1905	115	1898	38.0%	1.44 [1.14, 1.81]	2006
**Subtotal (95% CI)**		**1905**		**1898**	**38.0%**	**1.44 [1.14, 1.81]**	
Total events	166		115				
Heterogeneity: Not applicable							
Test for overall effect: Z = 3.11 (P = 0.002)							
**8.1.2 More statins/ezetimibe vs. Less statins/ezeimibe**							
TST [10]	103	1092	82	1106	26.9%	1.27 [0.96, 1.68]	2020
**Subtotal (95% CI)**		**1092**		**1106**	**26.9%**	**1.27 [0.96, 1.68]**	
Total events	103		82				
Heterogeneity: Not applicable							
Test for overall effect: Z = 1.70 (P = 0.09)							
**8.1.3 PCSK9 inhibitors plus statins vs. Placebo plus statins**							
FOURIER [2]	114	1493	106	1475	35.2%	1.06 [0.82, 1.37]	2020
**Subtotal (95% CI)**		**1493**		**1475**	**35.2%**	**1.06 [0.82, 1.37]**	
Total events	114		106				
Heterogeneity: Not applicable							
Test for overall effect: Z = 0.47 (P = 0.64)							
**Total (95% CI)**		**4490**		**4479**	**100.0%**	**1.26 [1.09, 1.46]**	
Total events	383		303				
Heterogeneity: Chi² = 3.01, df = 2 (P = 0.22); I² = 34%							
Test for overall effect: Z = 3.14 (P = 0.002)							
Test for subgroup differences: Chi² = 3.01, df = 2 (P = 0.22), I² = 33.5%							

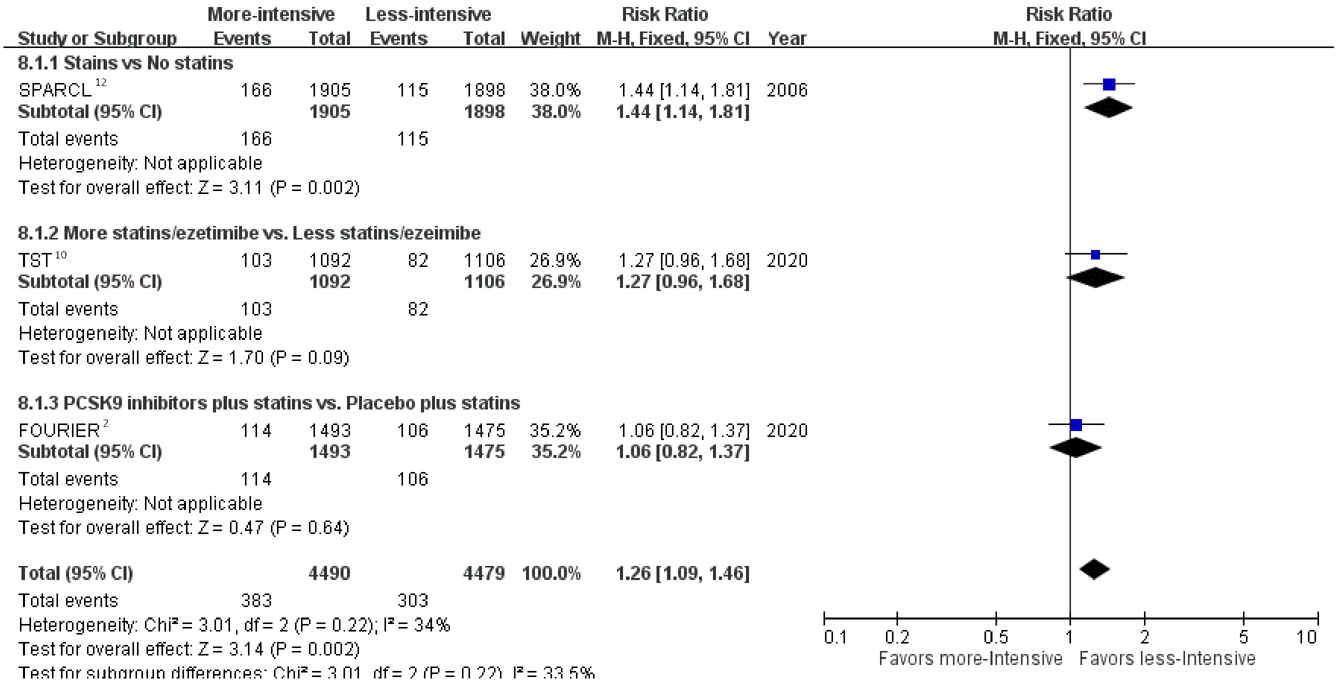

© 2022 Lee M et al. *JAMA Neurology*.

eFigure 9. Cognitive Adverse Events

Legends: Relative risk with 95% confidence interval of cognitive adverse events in more intensive vs less intensive LDL-C lowering with statin-based therapies in patients with stroke

	More-intensive		Less-intensive			Risk Ratio	
**Study or Subgroup**	**Events**	**Total**	**Events**	**Total**	**Weight**	**M-H, Fixed, 95% CI**	**Year**
**9.1.1 Statins vs. No statins**							
J-STARS[5]	33	793	33	785	38.3%	0.99 [0.62, 1.59]	2015
**Subtotal (95% CI)**		**793**		**785**	**38.3%**	**0.99 [0.62, 1.59]**	
Total events	33		33				
Heterogeneity: Not applicable							
Test for overall effect: Z = 0.04 (P = 0.97)							
**9.1.2 PCSK9 inhibitors plus statins vs. Placebo plus statins**							
FOURIER[2]	53	2686	53	2651	61.7%	0.99 [0.68, 1.44]	2020
**Subtotal (95% CI)**		**2686**		**2651**	**61.7%**	**0.99 [0.68, 1.44]**	
Total events	53		53				
Heterogeneity: Not applicable							
Test for overall effect: Z = 0.07 (P = 0.95)							
**Total (95% CI)**		**3479**		**3436**	**100.0%**	**0.99 [0.74, 1.33]**	
Total events	86		86				
Heterogeneity: Chi² = 0.00, df = 1 (P = 0.99); I² = 0%							
Test for overall effect: Z = 0.08 (P = 0.94)							
Test for subgroup differences: Chi² = 0.00, df = 1 (P = 0.99), I² = 0%							

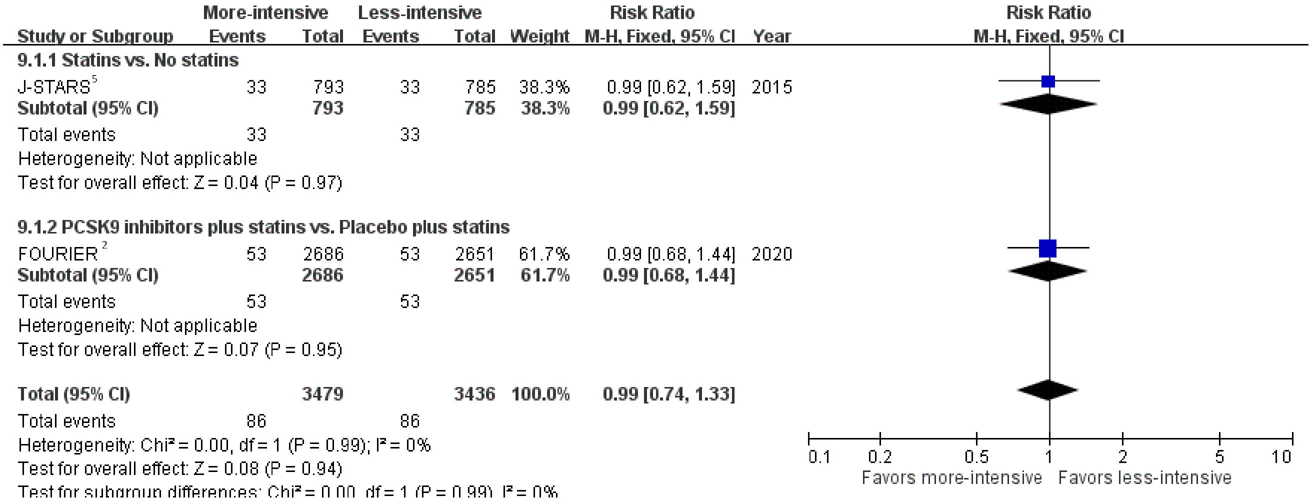

© 2022 Lee M et al. *JAMA Neurology*.

eFigure 10. Recurrent Stroke

Legends: Relative risk with 95% confidence interval of recurrent stroke in more intensive vs less intensive LDL-C lowering with statin-based therapies in patients with ischemic stroke as an entry event

Study or Subgroup	More intensive Events	More intensive Total	Less intensive Events	Less intensive Total	Weight	Risk Ratio M-H, Fixed, 95% CI	Year
**10.1.1 Statins vs. No statins**							
SPARCL[13]	141	1075	180	1083	45.4%	0.79 [0.64, 0.97]	2006
Yakusevich et al.[11]	13	86	14	97	3.3%	1.05 [0.52, 2.10]	2012
J-STARS[5]	91	793	95	785	24.2%	0.95 [0.72, 1.24]	2015
**Subtotal (95% CI)**		**1954**		**1965**	**73.0%**	**0.85 [0.73, 1.00]**	
Total events	245		289				
Heterogeneity: Chi² = 1.48, df = 2 (P = 0.48); I² = 0%							
Test for overall effect: Z = 1.96 (P = 0.05)							
**10.1.2 More statins/ezetimibe vs Less statins/ezetimibe**							
PODCAST[8]	2	39	1	38	0.3%	1.95 [0.18, 20.61]	2017
**Subtotal (95% CI)**		**39**		**38**	**0.3%**	**1.95 [0.18, 20.61]**	
Total events	2		1				
Heterogeneity: Not applicable							
Test for overall effect: Z = 0.55 (P = 0.58)							
**10.1.3 PCSK9 inhibitors plus statins vs. Placebo plus statins**							
FOURIER[2]	95	2686	105	2651	26.8%	0.89 [0.68, 1.17]	2020
**Subtotal (95% CI)**		**2686**		**2651**	**26.8%**	**0.89 [0.68, 1.17]**	
Total events	95		105				
Heterogeneity: Not applicable							
Test for overall effect: Z = 0.81 (P = 0.42)							
**Total (95% CI)**		**4679**		**4654**	**100.0%**	**0.87 [0.76, 0.99]**	
Total events	342		395				
Heterogeneity: Chi² = 2.02, df = 4 (P = 0.73); I² = 0%							
Test for overall effect: Z = 2.05 (P = 0.04)							
Test for subgroup differences: Chi² = 0.53, df = 2 (P = 0.77), I² = 0%							

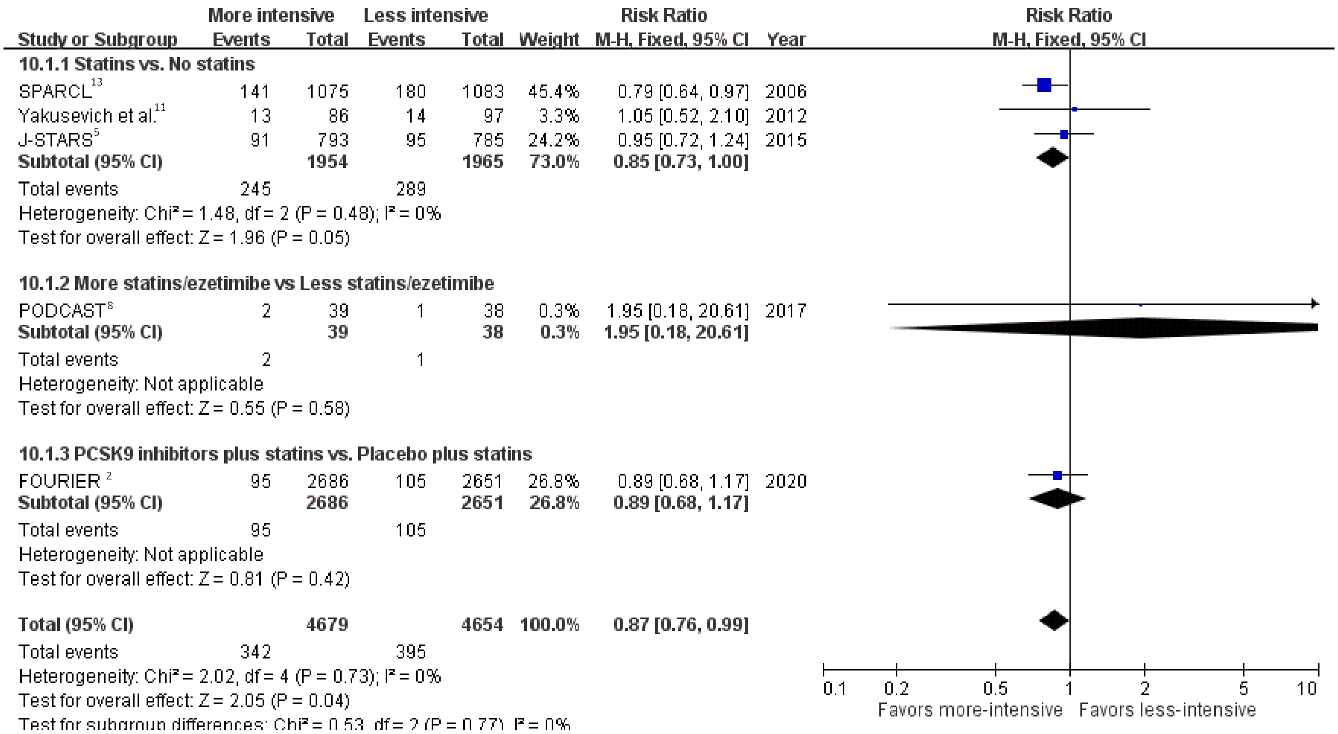

© 2022 Lee M et al. *JAMA Neurology*.

eFigure 11. Recurrent Stroke

Legends: Relative risk with 95% confidence interval of recurrent stroke in more intensive vs less intensive LDL-C lowering with statin-based therapies in patients with stroke and taking statins in both arms

Study or Subgroup	More-intensive Events	More-intensive Total	Less-intensive Events	Less-intensive Total	Weight	Risk Ratio M-H, Fixed, 95% CI	Year
**11.1.1 More statins/ezetimibe vs. Less statins/ezetimibe**							
IMPROVE-IT[4]	29	336	48	346	15.4%	0.62 [0.40, 0.96]	2017
PODCAST[8]	2	39	1	38	0.3%	1.95 [0.18, 20.61]	2017
TST[10]	103	1430	126	1430	41.1%	0.82 [0.64, 1.05]	2020
**Subtotal (95% CI)**		**1805**		**1814**	**56.9%**	**0.77 [0.62, 0.96]**	
Total events	134		175				
Heterogeneity: Chi² = 1.74, df = 2 (P = 0.42); I² = 0%							
Test for overall effect: Z = 2.37 (P = 0.02)							
**11.1.2 PCSK9 inhibitors plus statins vs. Placebo plus statins**							
ODYSSEY OUTCOMES[7]	25	477	26	467	8.6%	0.94 [0.55, 1.61]	2019
FOURIER[2]	95	2686	105	2651	34.5%	0.89 [0.68, 1.17]	2020
**Subtotal (95% CI)**		**3163**		**3118**	**43.1%**	**0.90 [0.71, 1.15]**	
Total events	120		131				
Heterogeneity: Chi² = 0.03, df = 1 (P = 0.86); I² = 0%							
Test for overall effect: Z = 0.83 (P = 0.41)							
**Total (95% CI)**		**4968**		**4932**	**100.0%**	**0.83 [0.70, 0.97]**	
Total events	254		306				
Heterogeneity: Chi² = 2.69, df = 4 (P = 0.61); I² = 0%							
Test for overall effect: Z = 2.30 (P = 0.02)							
Test for subgroup differences: Chi² = 0.91, df = 1 (P = 0.34), I² = 0%							

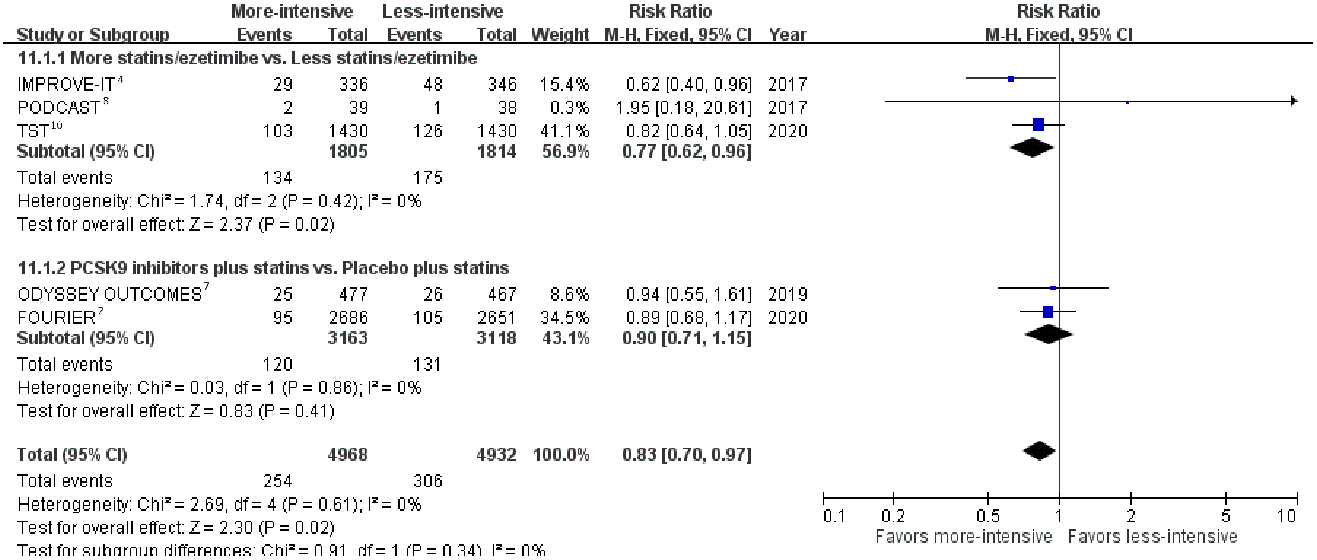

© 2022 Lee M et al. *JAMA Neurology*.

eFigure 12. Meta-regression

Legends: Meta-regression of included trials to explore the relation between degree of LDL-C lowering and recurrent stroke rate

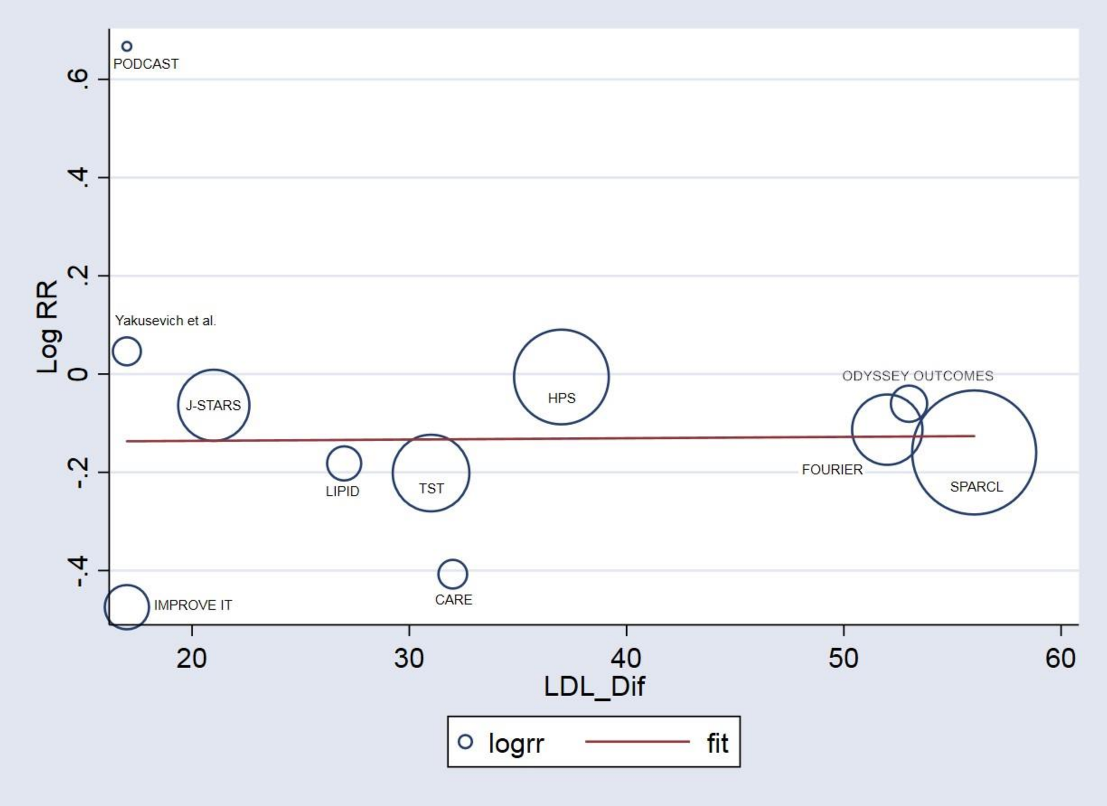

© 2022 Lee M et al. *JAMA Neurology*.

eFigure 13. Subgroup Analyses

Legends: Subgroup analyses based on the characteristics of trials

Subgroups	No. of studies	Risk ratio (95% CI)	P value for subgroup differences
**Baseline LDL-C**			
> 100 mg/dl	6	0.89 (0.80- 0.98)	0.71
< 100 mg/dl	5	0.85 (0.69- 1.04)	
**Degree of LDL-C decrease, mg/dL**			
< 39 mg/dl (1 mmol/L)	8	0.89 (0.79- 1.00)	0.78
> 39 mg/dl (1 mmol/L)	3	0.87 (0.76- 0.99)	
**Degree of LDL-C decrease, %**			
< 30%	8	0.89 (0.79- 1.00)	0.89
30-49%	1	0.85 (0.73- 0.99)	
≧50%	2	0.90 (0.71- 1.15)	
**Evidence of atherosclerosis**			
All patients having evidence of atherosclerosis	7	0.79 (0.69- 0.91)	0.04
Most patients not having evidence of atherosclerosi	5	0.95 (0.85- 1.07)	
**Study duration**			
< 3 years	4	0.92 (0.73- 1.16)	0.65
> 3 years	7	0.87 (0.79- 0.96)	
**Sample size**			
< 200	3	0.87 (0.54- 1.40)	0.58
200-1000	3	0.76 (0.57- 1.01)	
> 1000	5	0.89 (0.81- 0.98)	
**Study design of original tirals**			
All patients having stroke or TIA	5	0.87 (0.77- 0.97)	0.74
Subgroup of patients having stroke	6	0.89 (0.78- 1.03)	
**Coronary artery disease**			
All patients having concomitant coronary artery disease	4	0.75 (0.57- 0.97)	0.42
All patients not having concomitant coronary artery disease	4	0.91 (0.81- 1.02)	
Some patients having concomitant coronary artery disease	3	0.88 (0.76- 1.02)	

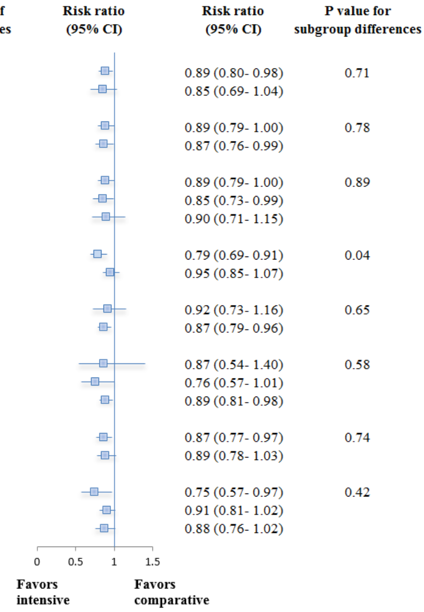

© 2022 Lee M et al. *JAMA Neurology*.

eFigure 14. Publication Bias

Legends: Trim-and-fill analysis for included trials to explore potential publication bias

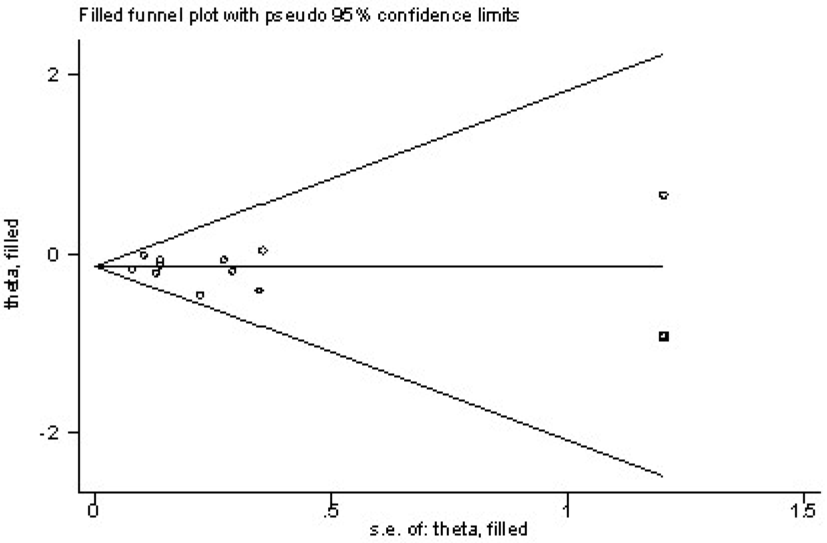

© 2022 Lee M et al. *JAMA Neurology*.

eReferences.

1. Plehn JF, Davis BR, Sacks FM, et al. Reduction of stroke incidence after myocardial infarction with pravastatin: the Cholesterol and Recurrent Events (CARE) study. The Care Investigators. Circulation. 1999;99(2):216-223.
2. Giugliano RP, Pedersen TR, Saver JL, et al. Stroke Prevention With the PCSK9 (Proprotein Convertase Subtilisin-Kexin Type 9) Inhibitor Evolocumab Added to Statin in High-Risk Patients With Stable Atherosclerosis. Stroke. 2020;51(5):1546-1554.
3. Collins R, Armitage J, Parish S, Sleight P, Peto R, Heart Protection Study Collaborative G. Effects of cholesterol-lowering with simvastatin on stroke and other major vascular events in 20536 people with cerebrovascular disease or other high-risk conditions. Lancet. 2004;363(9411):757-767.
4. Bohula EA, Wiviott SD, Giugliano RP, et al. Prevention of Stroke with the Addition of Ezetimibe to Statin Therapy in Patients With Acute Coronary Syndrome in IMPROVE-IT (Improved Reduction of Outcomes: Vytorin Efficacy International Trial). Circulation. 2017;136(25):2440-2450.
5. Hosomi N, Nagai Y, Kohriyama T, et al. The Japan Statin Treatment Against Recurrent Stroke (J-STARS): A Multicenter, Randomized, Open-label, Parallel-group Study. EBioMedicine. 2015;2(9):1071-1078.
6. White HD, Simes RJ, Anderson NE, et al. Pravastatin therapy and the risk of stroke. N Engl J Med. 2000;343(5):317-326.
7. Jukema JW, Zijlstra LE, Bhatt DL, et al. Effect of Alirocumab on Stroke in ODYSSEY OUTCOMES. Circulation. 2019;140(25):2054-2062.
8. Bath PM, Scutt P, Blackburn DJ, et al. Intensive versus Guideline Blood Pressure and Lipid Lowering in Patients with Previous Stroke: Main Results from the Pilot 'Prevention of Decline in Cognition after Stroke Trial' (PODCAST) Randomised Controlled Trial. PLoS One. 2017;12(1):e0164608.
9. Amarenco P, Bogousslavsky J, Callahan A, 3rd, et al. High-dose atorvastatin after stroke or transient ischemic attack. N Engl J Med. 2006;355(6):549-559.
10. Amarenco P, Kim JS, Labreuche J, et al. A Comparison of Two LDL Cholesterol Targets after Ischemic Stroke. N Engl J Med. 2020;382(1):9.
11. Yakusevich VV, Malygin AY, Lychenko SV, Petrochenko AS, Kabanov AV. The efficacy of high-dose simvastatin in acute period of ischemic stroke. Rational Pharmacother Card. 2012;8:4-16.

© 2022 Lee M et al. *JAMA Neurology*.

12. Waters DD, Ho JE, DeMicco DA, et al. Predictors of new-onset diabetes in patients treated with atorvastatin: results from 3 large randomized clinical trials. J Am Coll Cardiol. 2011;57(14):1535-1545.
13. Amarenco P, Benavente O, Goldstein LB, et al. Results of the Stroke Prevention by Aggressive Reduction in Cholesterol Levels (SPARCL) trial by stroke subtypes. Stroke. 2009;40(4):1405-1409.

© 2022 Lee M et al. *JAMA Neurology*.
